# Role of PRC2 in the stochastic expression of Aire target genes and development of mimetic cells in the thymus

**DOI:** 10.1084/jem.20240817

**Published:** 2025-04-17

**Authors:** Minoru Matsumoto, Masaki Yoshida, Takeshi Oya, Koichi Tsuneyama, Mitsuru Matsumoto, Hideyuki Yoshida

**Affiliations:** 1 Department of Molecular Pathology, Tokushima University Graduate School of Biomedical Sciences, Tokushima, Japan; 2 YCI Laboratory for Immunological Transcriptomics, RIKEN Center for Integrative Medical Science, Yokohama, Japan; 3 Department of Pathology and Laboratory Medicine, Tokushima University Graduate School of Biomedical Sciences, Tokushima, Japan; 4 Division of Molecular Immunology, Institute for Enzyme Research, Tokushima University, Tokushima, Japan; 5 Department of Endocrinology, Diabetes and Metabolism, Kitasato University School of Medicine, Sagamihara, Japan

## Abstract

The transcriptional targets of Aire and the mechanisms controlling their expression in medullary thymic epithelial cells (mTECs) need to be clarified to understand Aire’s tolerogenic function. By using a multi-omics single-cell approach coupled with deep scRNA-seq, we examined how Aire controls the transcription of a wide variety of genes in a small fraction of Aire-expressing cells. We found that chromatin repression by PRC2 is an important step for Aire to achieve stochastic gene expression. Aire unleashed the silenced chromatin configuration caused by PRC2, thereby increasing the expression of its functional targets. Besides this preconditioning for Aire’s gene induction, we demonstrated that PRC2 also controls the composition of mTECs that mimic the developmental trait of peripheral tissues, i.e., mimetic cells. Of note, this action of PRC2 was independent of Aire and it was more apparent than Aire. Thus, our study uncovered the essential role of polycomb complex for Aire-mediated promiscuous gene expression and the development of mimetic cells.

## Teaser

This study highlights the contribution of PRC2 to Aire-mediated stochastic gene expression and the development of mimetic cells through various single-cell approaches. The genomics data utilized in this work serve as a valuable resource for advancing research on Aire.

## Graphical Abstract

**Figure:**
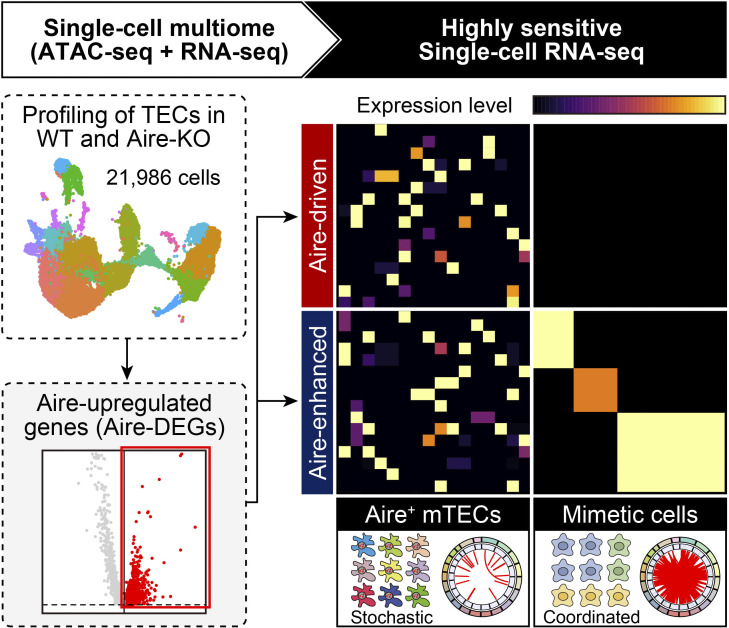

## Introduction

In the thymus, T cells that are autoreactive to self-antigens are either eliminated by negative selection or develop into regulatory T cells (Tregs), depending on their avidities for the cognate self-antigens, to prevent the autoimmunity (Klein et al., 2014; Stritesky et al., 2012). For this purpose, medullary thymic epithelial cells (mTECs) play a pivotal role by expressing and presenting the immunological self, which, in principle, encompasses all the self-antigens expressed by peripheral parenchymal organs (Kyewski and Klein, 2006). Aire in mTECs has been demonstrated to control the expression of self-antigens, and a deficiency of Aire results in the development of organ-specific autoimmune diseases due, at least in part, to a defect in the expression of tissue-restricted self-antigens (TRAs) (Březina et al., 2022; Abramson et al., 2010; Anderson et al., 2002). How Aire controls a wide variety of genes in the mTECs, including TRAs, has been a subject of intense research (Matsumoto, 2011).

Until the advent of single-cell technologies, Aire’s action on the transcriptome had been studied through comparisons between wild-type (WT) and Aire-deficient mice (Aire-KO) using bulk mature mTECs marked with CD80^high^MHC class II (MHC-II)^high^, in which Aire-expressing cells are enriched. Genes down-regulated in Aire-deficient mTECs, including many TRA genes, have been called “Aire-induced genes” (Březina et al., 2022; Abramson et al., 2010; Anderson et al., 2002; Sansom et al., 2014). However, these Aire-induced genes defined by bulk RNA sequencing (RNA-seq) analysis do not necessarily imply that they are bona fide transcriptional targets of Aire. Because Aire controls the developmental program of mTECs (Yano et al., 2008), the absence of Aire would also change mTEC composition, thereby resulting in altered gene expression from mTECs. Consistent with this idea, we have demonstrated the alteration in the cluster formation of mTECs from Aire-KO by single-cell RNA-seq (scRNA-seq) analysis (Nishijima et al., 2022). However, the exact property of the two types of Aire-induced genes (i.e., genes directly controlled by Aire and genes whose levels are affected by the altered composition of mTECs) has not been demonstrated. Clarifying these distinct gene regulatory mechanisms is crucial for understanding how mTECs mediate immune tolerance with the aid of Aire.

In the present study, our first aim was to define the precise subpopulations of mTECs and to reveal the impact of Aire on their composition. This was accomplished by employing an analysis of gene expression (scRNA-seq) coupled with the chromatin state (scATAC-seq) from the same single TECs (scMulti-seq) (Hao et al., 2021; Delacher et al., 2021). The scMulti-seq identified mTEC subpopulation at unprecedentedly high resolution with their determinant transcription factors (TFs), including the mimetic cells that employ the same TFs for their development as those in the corresponding peripheral organs (Michelson et al., 2022; Givony et al., 2023). Consequently, the scMulti-seq analysis revealed the exact outcome of Aire deficiency on the development of mTEC subpopulations, resulting in the reduction of some mimetic cell clusters. When we focused on Aire’s functional targets by isolating the up-regulated genes in the primary Aire-expressing cells from WT compared with their counterparts from Aire-KO (i.e., Aire-differentially expressed genes [“Aire-DEGs” thereafter]), we noticed that there were two distinct types of Aire-DEGs. In the first type, Aire-DEGs were confined to the primary Aire-expressing cells, and their induction was dependent on Aire. In the second type, Aire-DEGs were expressed not only from the Aire-expressing cells but also from other mimetic cell clusters. Of note, these mimetic cell clusters expressed Aire-DEGs in an Aire-independent manner. While each type of gene may contribute differently to the formation of Aire-induced genes when bulk mature mTECs are analyzed, scMulti-seq enabled us to accurately dissect the regulatory mechanisms for Aire’s gene induction by focusing on the former type. In this regard, although scMulti-seq is extremely powerful for profiling the cells, we admit that the profiling depth in each cell might be insufficient to fully appreciate the nature of Aire’s gene control. To circumvent this point, we introduced the highly sensitive scRNA-seq method, RamDA-seq, to demonstrate the stochastic activation of Aire’s gene induction in a small fraction of cells, reflecting the promiscuous nature of TRA gene expression. Furthermore, we found that the expression of Aire targets was progressively suppressed during the maturation of mTECs, and this was associated with the enhancement of the repressive H3K27me3 chromatin mark regulated by the polycomb repressive complex 2 (PRC2). Aire expression in the mature Aire-expressing stage unleashed the silenced chromatin configuration caused by PRC2, thereby increasing the expression of Aire targets. Additionally, we demonstrated that PRC2 controls the development of mimetic cells independently of Aire, as revealed by the RamDA-seq analysis of mTECs deficient for both PRC2 and Aire. Collectively, our study unveiled the essential role of the polycomb complex in the promiscuous gene expression from mTECs controlled by Aire and in the development of mimetic cells.

## Results

### Multiple mTEC subpopulations harboring distinct transcriptome and TF activities

**Figure S1.**
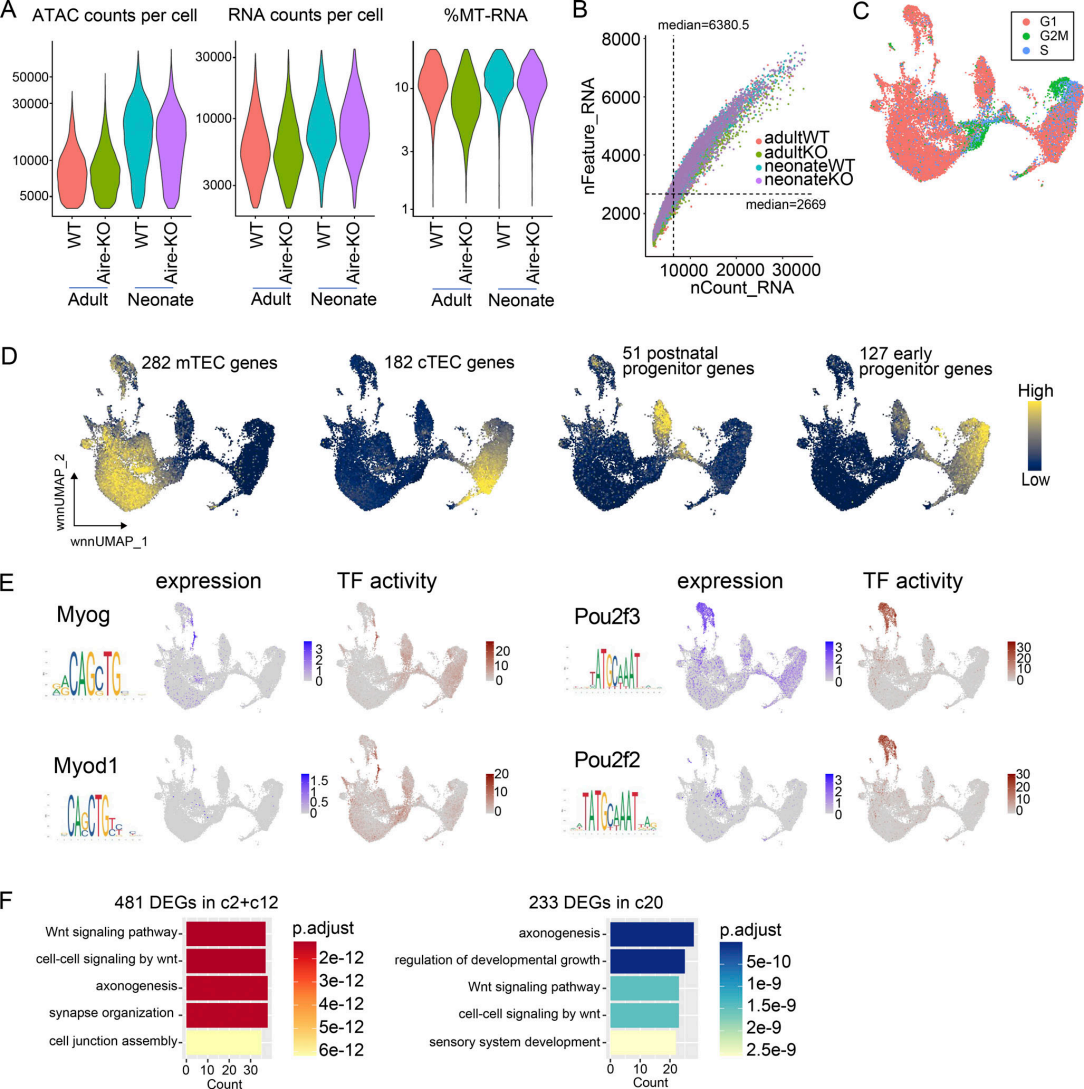
**Evaluation and supplementary analysis of scMulti-seq data.** Related to Fig. 1. **(A)** Quality assessment of samples employed for the scMulti-seq analysis. **(B)** Number of RNA molecules (nCount_RNA) and genes (nFeature_RNA) detected in each cell, with a median value of 2,669 genes per cell. **(C)** UMAP visualization displaying classified cell cycle phases predicted by Seurat based on gene expression patterns. **(D)** UMAP color-coded based on the mean expression levels of indicated gene sets in each cell. **(E)** Example plots showing the discrepancy between the gene expressions evaluated by scRNA-seq and motif activities estimated by scATAC-seq for certain TFs due to similar binding motifs among the same TF family. **(F)** GO analysis employing DEGs (average logFC > 0.5 and P value <1 × 10^−5^ by the Wilcoxon rank-sum test) in the indicated clusters.

**Figure 1.**
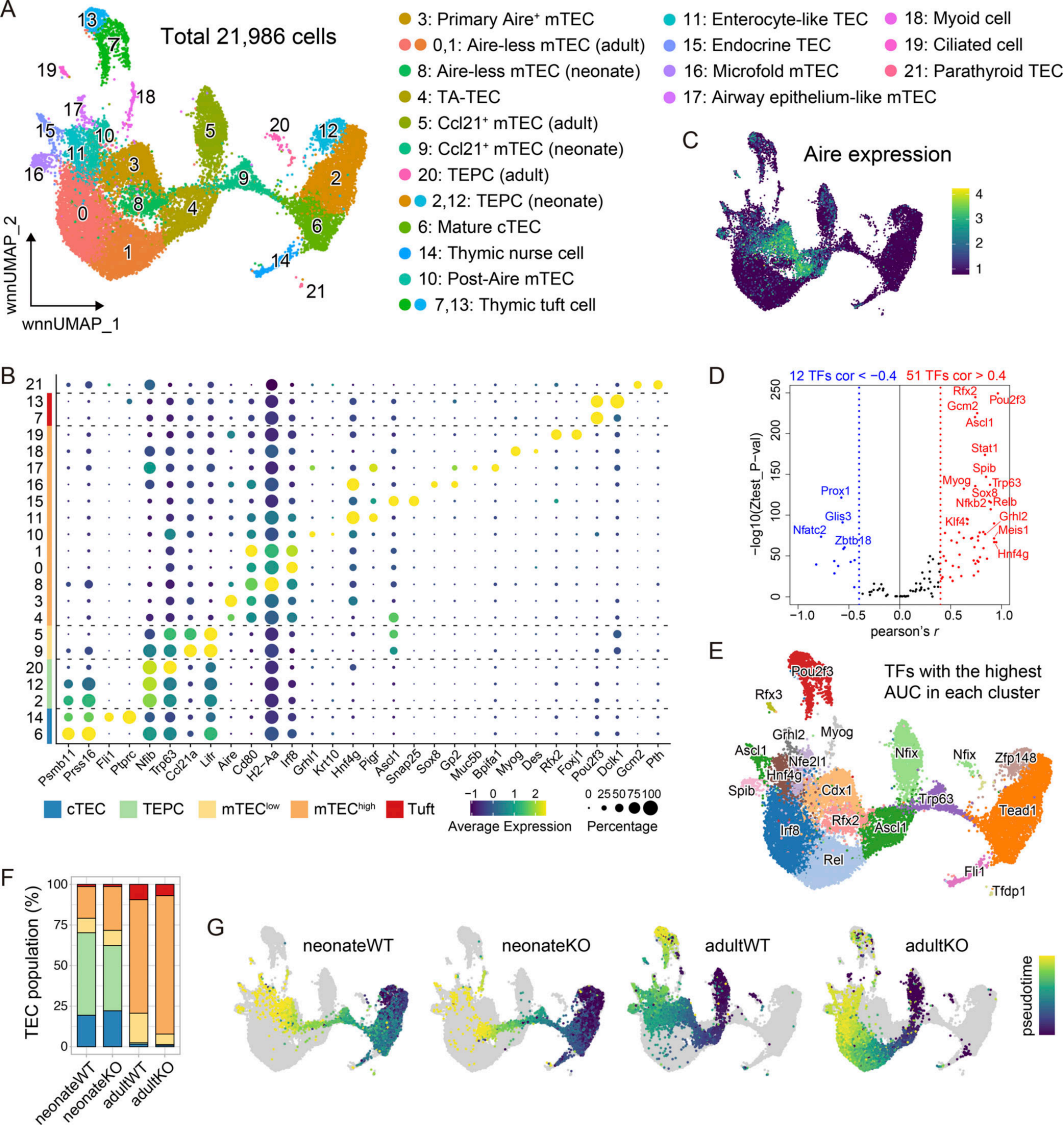
**Multiple TEC subpopulations impacted by age and Aire. (A)** UMAP visualization of the four samples analyzed by scMulti-seq. Each dot indicates a single cell (see also Table S1). **(B)** Dot plot showing the expression levels for respective genes. TEC subpopulations are indicated by color coding on the left bar. **(C)** UMAP color-coded based on the Aire expression level (natural log+1). **(D)** Correlations between the expression level of a TF and its estimated activity based on chromatin accessibility. P values by permutation tests. **(E)** UMAP color-coded according to the representative TFs showing the highest AUC in each cluster. **(F)** Proportion of TEC subpopulations in each sample. Color coding of TEC subpopulations is same as B. **(G)** UMAPs color-coded based on the pseudotime analysis in each sample.

We utilized FACS-sorted CD45^−^EpCAM^+^ TECs from adults and neonates of both WT and Aire-KO genotypes to identify diverse TEC subpopulations. After quality filtering (Fig. S1, A and B), we obtained transcriptomes and chromatin profiles from a total of 21,986 cells (Table S1). The integration of RNA expression and chromatin profiles using weighted nearest neighbor (WNN) analysis (Hao et al., 2021) resulted in the emergence of 22 clusters (Fig. 1 A). Each cluster exhibited distinct characteristics, as demonstrated by its specific gene expression profiles (Fig. 1 B and Table S1). Based on the expression levels of Cd80 and MHC-II (Fig. 1 B), we considered clusters in the left half of Fig. 1 A (c0, c1, c3, c4, c8, c10, c11, c15–c19) corresponded to CD80^high^MHC-II^high^ (mTEC^high^). We identified c3 in mTEC^high^, which existed only in the WT, as a primary Aire-expressing cluster (Fig. 1 C and Table S1). While the expression of Aire was also high in part of c4, this population was in the active cell cycle (Fig. S1 C) and most likely represented a transit-amplifying TEC (TA-TEC) as previously reported (Baran-Gale et al., 2020; Nishijima et al., 2022; Wells et al., 2020; Miyao et al., 2022). Among mTEC^high^ clusters, we considered clusters in the upper left of Fig. 1 A (c7, c10, c11, c13, c15–c19) corresponded to mimetic cells according to their characteristic gene signatures (Fig. 1 B and Table S1). These identifications were consistent with previously reported gene sets (Nusser et al., 2022) (Fig. S1 D), confirming that our data accurately represented the TEC subpopulations.

While comparing the expression levels and TF activities, we observed some discrepancies among certain TFs. These discrepancies included cases with similar motifs within the same TF family, as exemplified by Myod1 (cf. Myog) and Pou2f2 (cf. Pou2f3) (Fig. S1 E). Therefore, we took advantage of scMulti-seq, focusing on TFs that exhibited a significant correlation between their expression levels and TF activities, to determine signature TFs for each cluster (Fig. 1, D and E). This approach effectively captured unique TFs beyond the TF family level within each mimetic cell cluster. Thus, our multi-omics analysis revealed TEC subpopulations with unique transcriptomes and signature TFs at significantly high resolution.

### Aire and age dependency of mTEC subpopulations

Each sample showed a distinct composition, demonstrating the subpopulations produced in an Aire- and age-dependent manner (Fig. 1 F and Table S1). We detected unique clusters (c2 and c12) in neonate samples expressing early progenitor genes together with cortical TEC (cTEC) genes (Fig. 1 A and Fig. S1 D), which likely correspond to thymic epithelial progenitor cells (TEPCs) (Ohigashi et al., 2013; Baik et al., 2013; Nusser et al., 2022). We also identified a small cluster in the adults (c20) exhibiting TEPC signatures with the high expression of early progenitor genes (Fig. 1 A and Fig. S1 D) and activation of the Wnt signaling pathway, similar to the clusters c2 and c12 in neonates (Fig. S1 F). The identification of putative TEPCs in adults was most likely achieved by introducing the samples from neonates and adults to the scMulti-seq analysis because c20 in adults was too small to be identified as a cluster without referring to the resembling clusters c2 and c12.

Then, we evaluated the developmental trajectory of mTEC clusters in each sample by the pseudotime analysis (Fig. 1 G). As the scMulti-seq pipeline utilizes isolated nuclei, which lack cytoplasmic RNA, we proceeded with the pseudotime analysis employing the ATAC-seq data. We set c12 as the root cells for neonates, considering their active cell cycle (Fig. S1 C), presumably reflecting the highest capacity as progenitor cells. Meanwhile, we set c5 (mTEC^low^) as the root cells for adults because mTEC maturation proceeds from mTEC^low^ to mTEC^high^ (Gray et al., 2007). Tracking the mTEC developmental trajectory in the neonates, c9 proceeded to c4 in both WT and Aire-KO, followed by c3 and c8 in WT and Aire-KO, respectively (Fig. 1 G). In the adults, c5 proceeded to c4 in both WT and Aire-KO, followed by c3 in WT and c0/c1 in Aire-KO. Because c8 in neonates and c0/c1 in adults scarcely expressed Aire (Fig. 1 C), we considered them the counterpart clusters of the primary Aire-expressing cells from WT that were alternatively developed in Aire-KO (Aire-less mTECs).

### Two distinct types of Aire dependency in the transcriptome

**Figure 2.**
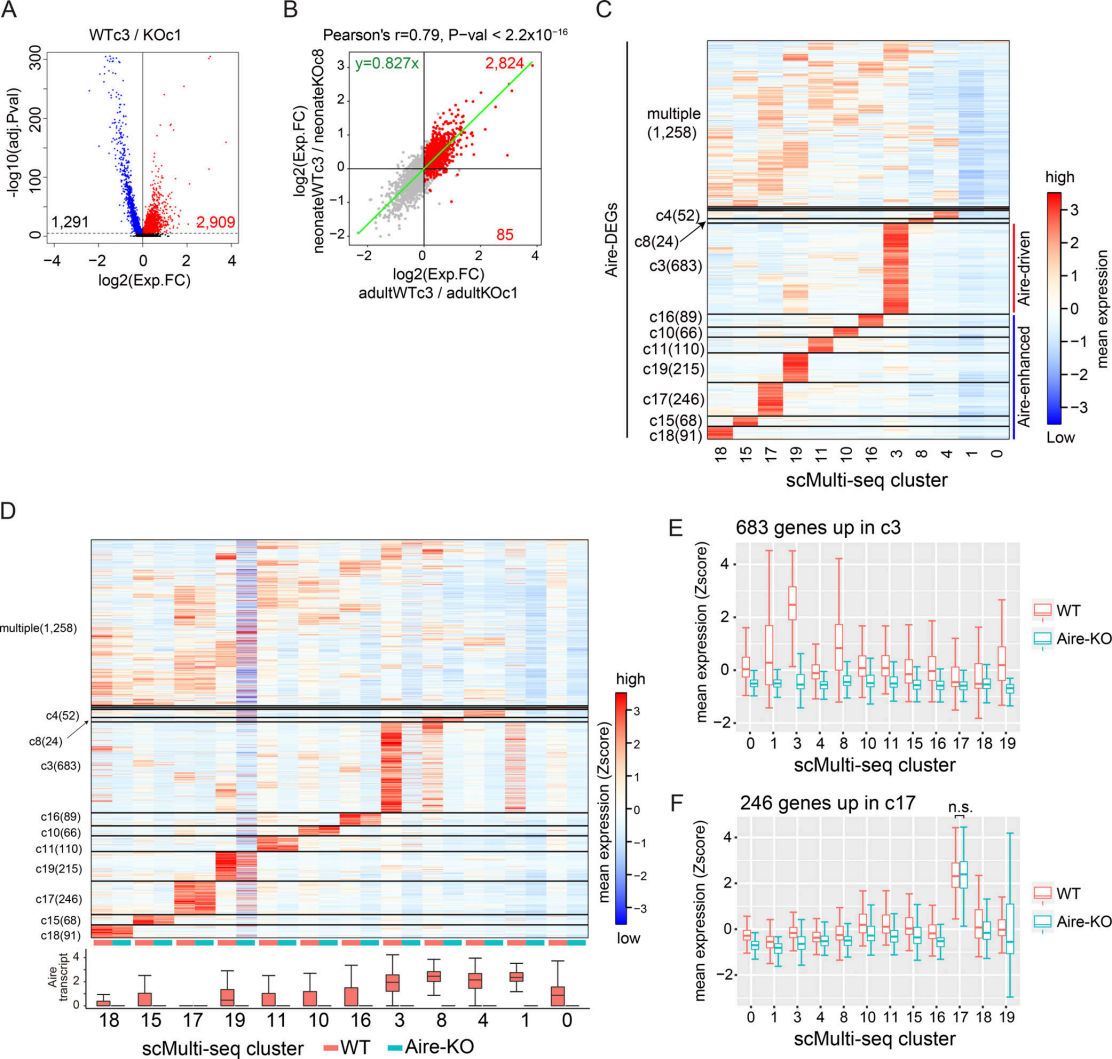
**Two distinct types of Aire dependency for Aire-DEGs. (A)** Volcano plot comparing the transcriptome of scMulti-seq WT/c3 and Aire-KO/c1, with Aire-DEGs highlighted in red. P values from the Wilcoxon rank-sum test were Bonferroni-corrected. **(B)** Comparisons of the gene induction by Aire in adults and neonates, with Aire-DEGs highlighted in red. The green trendline represents the SMA regression. Pearson’s correlation coefficient and P value by the F-test are indicated at the top. **(C)** Heatmap of Aire-DEG expression across mTEC^high^ clusters, represented by standardized expressions among the plotted clusters. Genes were grouped according to their expression patterns. **(D)** Heatmap of Aire-DEG expression across mTEC^high^ clusters, distinguishing WT and Aire-KO cells. The abundance of Aire transcripts in cells (natural log+1) is shown at the bottom. **(E and F)** Box plots representing the expression of Aire-DEGs predominantly up-regulated in the indicated cluster. P value in F was calculated by one-way ANOVA followed by Tukey’s test.

The transcriptional targets of Aire have been discussed by comparing the FACS-sorted mTEC^high^ fraction with bulk RNA-seq analysis. However, it is important to note that the mTEC^high^ fraction comprises several subpopulations besides Aire-expressing mTECs or Aire-less mTECs. This heterogeneity within the mTEC^high^ fraction would confound the estimation of Aire’s direct gene control. To precisely define genes controlled by Aire, we compared the gene expression from the primary Aire-expressing cells from WT (WT/c3) with that from the counterpart cells from Aire-KO (Aire-KO/c1) in adults. We selected Aire-KO/c1 as the authentic counterpart of WT/c3 because it was located next to its putative precursor c4 (TA-TEC) (Fig. 1 A) and contained much less WT cells compared with Aire-KO/c0 (Table S1). A total of 2,909 genes showed significantly higher expression in WT/c3 compared with those in Aire-KO/c1, whereas 1,291 genes showed lower expression in WT/c3 than in Aire-KO/c1 (Fig. 2 A). We focused on the up-regulated genes in WT/c3 and defined them as Aire-differentially expressed genes (Aire-DEGs in short) throughout this study. We observed a significant correlation for this Aire’s gene induction between adult and neonate samples (Fig. 2 B), confirming a consistent functionality of Aire irrespective of age.

To assess how Aire-DEGs are regulated within mTEC clusters, we first annotated Aire-DEGs to mTEC^high^ clusters revealed by our scMulti-seq analysis. Approximately one-quarter of Aire-DEGs (683 out of 2,909 genes) were expressed predominantly from a single cluster of c3 (i.e., Aire-expressing mTECs) (Fig. 2 C, middle portion). Interestingly, almost one-third of Aire-DEGs (a total of 885 genes out of 2,909) were also expressed at high levels from a single mTEC^high^ cluster other than c3 (Fig. 2 C, lower portion). When the expression levels of Aire-DEGs were compared between WT and Aire-KO, Aire-DEGs confined to c3 were significantly higher in WT (Fig. 2 D, middle portion, and Fig. 2 E). In contrast, expression levels of Aire-DEGs from other clusters than c3 were not so different between the WT and Aire-KO as exemplified by c17 (Fig. 2 D, lower portion, and Fig. 2 F). Thus, although Aire-DEGs were extracted as putative transcriptional targets of Aire by comparing the primary Aire-expressing cells (WT/c3) and its counterpart from Aire-KO (Aire-KO/c1), a subset of genes was also expressed from other mTEC^high^ clusters outside c3 in an Aire-independent manner. Because Aire was absolutely required for the induction of Aire-DEGs confined to WT/c3 (i.e., 683 genes in c3, Fig. 2 E), we termed these genes “Aire-driven genes.” In contrast, other Aire-DEGs were also expressed in other mTEC^high^ clusters in an Aire-independent manner (e.g., 246 genes in c17, Fig. 2 F). However, their overall expression within the total mTEC^high^ population was enhanced through their expression in Aire-expressing mTECs (WT/c3). We call them “Aire-enhanced genes” hereafter.

### Aire-driven genes are expressed from a small fraction of Aire-expressing cells

**Figure S2.**
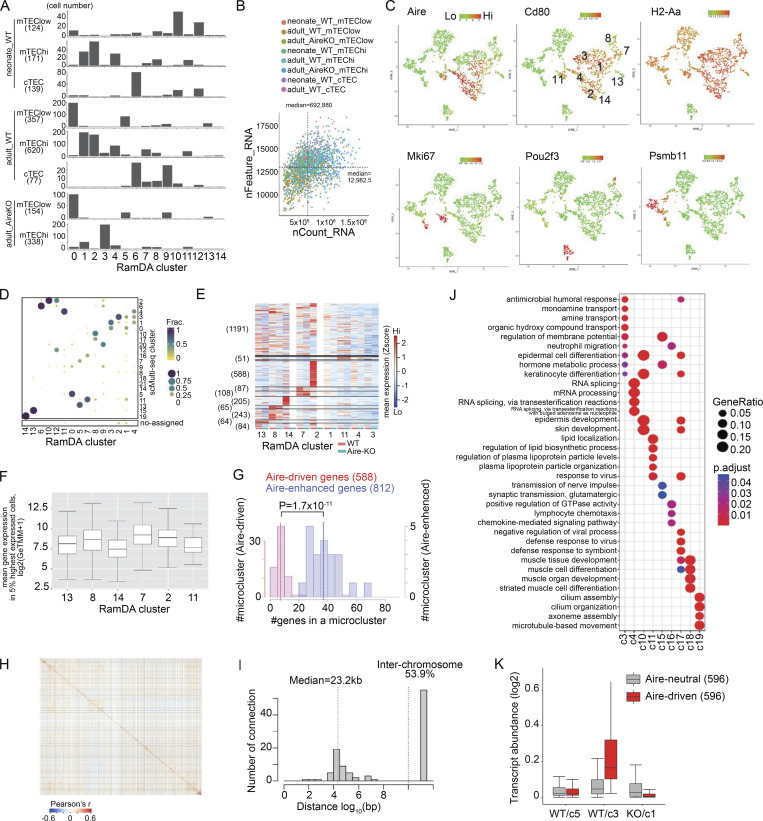
**Quality assessment and supplementary analysis of RamDA-seq data.** Related to Fig. 3. **(A)** Bar plots showing the cell numbers assigned to RamDA-seq clusters from each sample and gates for cell sorting. **(B)** Sequencing depth and the number of genes detected in each cell by RamDA-seq. Median: 12,983 genes detected per cell. **(C)** t-SNE plots color-coded for gene expression by RamDA-seq. Clusters corresponding to the mTEC^high^ are labeled in a plot for Cd80. **(D)** Cluster comparison between RamDA-seq (query) and scMulti-seq (reference). The size and color of the circles represent the proportion of cells from the query cluster corresponding to the reference cluster. **(E)** Expression of Aire-DEGs in mTEC^high^ clusters, distinguishing WT and Aire-KO cells. Genes are grouped according to the expression pattern across scMulti-seq clusters shown in Fig. 2 C (see also Fig. 3 D). **(F)** Box plot for the expression of Aire-DEGs predominantly up-regulated in each cluster. The mean expressions in log2(GeTMM+1) were calculated for the top 5% highest-expressing cells for each gene. **(G)** Histogram of the number of genes in a micro-cluster after clustering by affinity propagation (i.e., genes of intercorrelations). There were 68 micro-clusters with a median of 7 genes for Aire-driven genes and 21 micro-clusters with a median of 37 genes for Aire-enhanced genes. Dotted lines represent the median for each group, with a P value by the Wilcoxon rank-sum test (see also Fig. 3 G). **(H)** Gene-to-gene Pearson’s correlation coefficient calculated for 2,717 genes detected among 2,909 Aire-DEGs, using 463 cells within RamDA-WT/c2 and RamDA-Aire-KO/c3. **(I)** Histograms for the distance between highly correlated genes shown in H (Pearson’s r > 0.6, 102 connections). The median distance for the highly correlated genes located on the same chromosomes is indicated by a dotted line. **(J)** GO analysis of Aire-DEGs predominantly up-regulated in each cluster. P values from the hypergeometric distribution were Benjamini-Hochberg (BH)-corrected. **(K)** Box plot comparing expression levels of Aire-driven genes and Aire-neutral genes in indicated clusters.

Next, we focused on the expression patterns of Aire-driven genes and Aire-enhanced genes in mTEC^high^ clusters to elucidate the underlying mechanisms responsible for these two categories of Aire-DEGs. Although droplet-based scRNA-seq, including scMulti-seq, is suitable for clarifying the heterogeneous populations by profiling thousands of cells, the profiling depth in each cell may be insufficient to fully appreciate the nature of Aire’s gene control. To circumvent this issue, we employed the RamDA-seq method, which achieves much higher sensitivity than droplet-based scRNA-seq on a per-cell basis (Hayashi et al., 2018). We performed RamDA-seq analysis on FACS-sorted mTEC^low^ (CD45^−^EpCAM^+^Ly51^low^MHC-II^low^), mTEC^high^ (CD45^−^EpCAM^+^Ly51^low^MHC-II^high^), and cTECs (CD45^−^EpCAM^+^Ly51^high^) from adult WT, adult Aire-KO, and neonate WT samples (Fig. S2 A). As expected, RamDA-seq analysis provided much deeper transcriptomic information compared with scMulti-seq (12,983 by RamDA-seq versus 2,669 by scMulti-seq median detected genes per cell) (Fig. S1 B versus Fig. S2 B). RamDA-seq analysis also provided much deeper transcriptomic information when compared to the conventional scRNA-seq (3,184 by scRNA-seq median detected genes per cell) (Nishijima et al., 2022).

**Figure 3.**
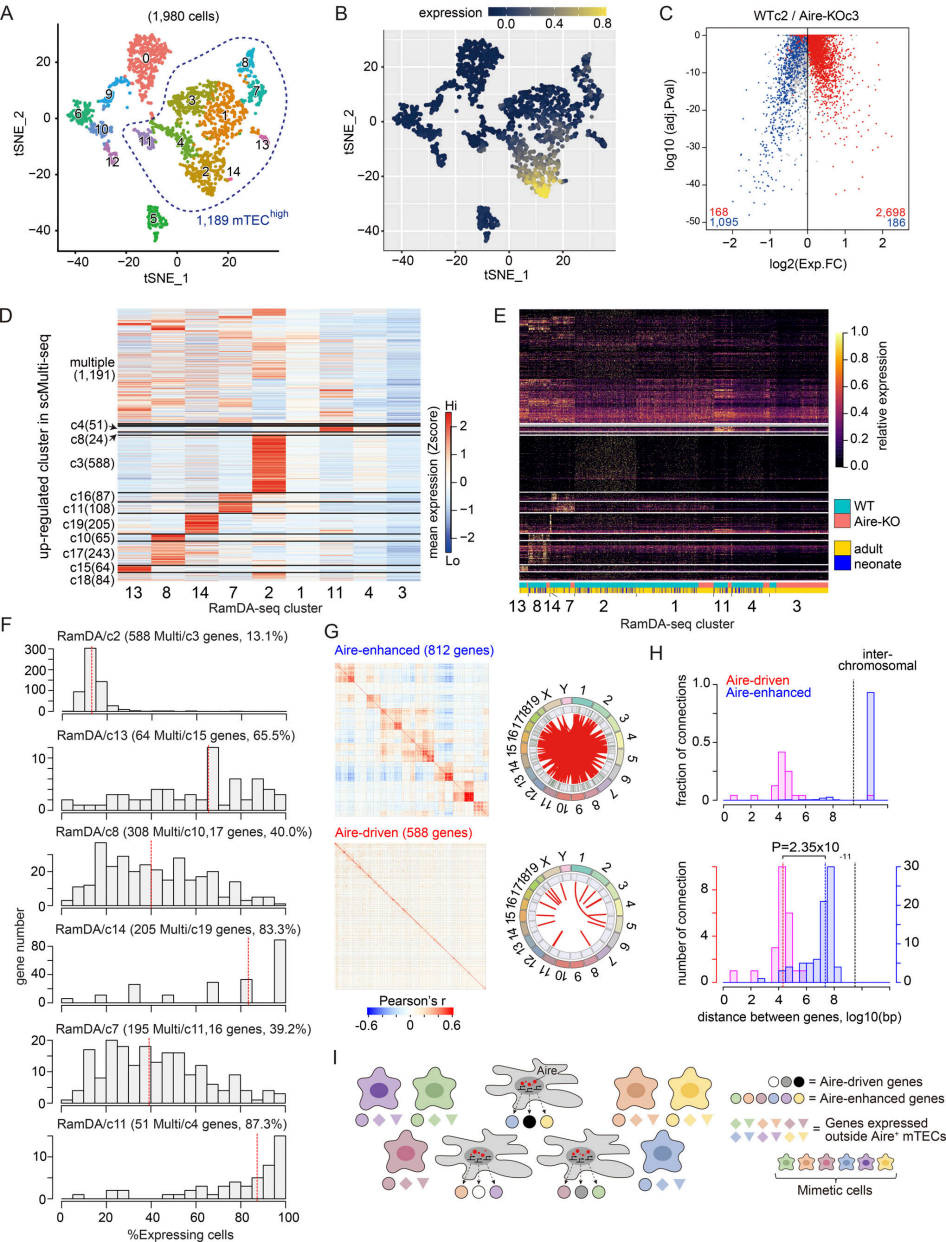
**Stochastic expression of Aire-induced transcripts revealed by deep single-cell analysis. (A)** t-SNE visualization of 1,980 TECs, color-coded by cluster assignment. **(B)** t-SNE plot color-coded for the mean expressions of 669 out of the 683 Aire-DEGs predominantly up-regulated in scMulti-seq/c3 (see also Fig. 2). **(C)** Volcano plot comparing RamDA-seq WT/c2 and Aire-KO/c3, with Bonferroni-adjusted P values from the Wilcoxon rank-sum test. Aire-DEGs defined by scMulti-seq in Fig. 2 A are highlighted in red. **(D)** Heatmap of 2,717 Aire-DEG expression across mTEC^high^ clusters, represented by standardized expressions. Genes were grouped as shown in Fig. 2. Genes expressed in fewer than five cells were excluded. **(E)** Expression of Aire-DEGs shown in D by individual cells. Expression levels are normalized to the 97.5th quantile and represented by color coding. Cell identities are indicated at the bottom. **(F)** Fractions of WT cells expressing Aire-DEGs predominantly up-regulated in each cluster. The median proportions of expressing cells are shown at the top and represented by red dashed lines in histograms. **(G)** Pearson’s correlation coefficient among Aire-enhanced genes (812 Aire-DEGs up-regulated in scMulti-seq-c4, scMulti-seq-c10, scMulti-seq-c11, scMulti-seq-c15-17, and scMulti-seq-c19 calculated with RamDA-c7, RamDA-c8, RamDA-c11, RamDA-c13, and RamDA-c14, top) and Aire-driven genes (588 Aire-DEGs predominantly up-regulated in scMulti-seq/c3 calculated with RamDA-c2 and RamDA-c3, bottom). Left: Gene-to-gene correlations after clustering by affinity propagation. Right: Circos plots indicating genomic positions for highly correlated genes (Pearson’s r > 0.6), with 1,157 and 24 connections by red lines for Aire-enhanced genes and Aire-driven genes, respectively (see also Fig. S2 H). **(H)** Histograms representing the distance between highly correlated genes within two Aire-DEG groups. Top: The relative frequency for the intra- and interchromosomal correlations (4.2% and 93.2% interchromosomal correlations for Aire-driven genes and Aire-enhanced genes, respectively; P = 7.7 × 10^−51^ by the chi-square test). Bottom: The frequency within the same chromosomes (total 23 connections for Aire-driven genes and 79 for Aire-enhanced genes; P = 2.4 × 10^−11^ by the Wilcoxon rank-sum test). **(I)** Illustration of two types of Aire-DEGs: Aire-driven genes and Aire-enhanced genes. Both types are directly induced by Aire’s transcriptional activity within Aire-expressing mTECs in a stochastic manner. Aire-enhanced genes are additionally expressed in mimetic cells in a coordinated manner.

We identified a total of 15 clusters (Fig. 3 A), including nine mTEC^high^ clusters (RamDA-c1, RamDA-c2, RamDA-c3, RamDA-c4, RamDA-c7, RamDA-c8, RamDA-c11, RamDA-c13, and RamDA-c14) based on the gates for cell sorting and the expression levels of Aire, Cd80, and MHC-II (Fig. 3 A and Fig. S2, A and C). While searching for clusters identical to c3 in scMulti-seq (scMulti-c3), we found that RamDA-c2 showed the highest expression of Aire-driven genes present in scMulti-c3 (Fig. 3 B). Furthermore, most cells in RamDA-c2 corresponded to scMulti-c3 by cell-to-cluster matching analysis based on the gene expression signature from each cluster (Fig. S2 D), indicating that RamDA-c2 and scMulti-c3 are well matched. Similarly, RamDA-c3 corresponded to the Aire-less mTECs (Aire-KO/c1 in scMulti-seq) because they were specific for Aire-KO and showed matched gene signatures (Fig. S2, A and D). Accordingly, the expression of 2,909 Aire-DEGs shown in Fig. 2 A was significantly higher in WT RamDA-c2 compared with that in the counterpart cluster of Aire-KO RamDA-c3 (Fig. 3 C and Table S2). Aire-enhanced genes in the other RamDA-seq clusters were also consistent with their expression in the matched scMulti-seq clusters (Fig. 3 D), and the lack of Aire in RamDA-seq clusters showed a similar pattern observed by the scMulti-seq analysis (compare Fig. S2 E with Fig. 2 D).

The high sensitivity of RamDA-seq analysis provided detailed gene expression patterns from individual cells. Aire-driven genes in RamDA-c2 from WT exhibited a scattered expression pattern in the heatmap (Fig. 3 E, middle portion), suggesting their stochastic expression for each gene. In contrast, a more homogeneous expression pattern was observed for Aire-enhanced genes in other clusters (Fig. 3 E, lower portion). Each Aire-driven gene was expressed in 13.1% of the cells in RamDA-c2, which was significantly lower than Aire-enhanced genes in other clusters (RamDA-c13, RamDA-c8, RamDA-c14, RamDA-c7, and RamDA-c11) (Tukey’s honestly significant difference (HSD) test, P value <2.2 × 10^−16^) (Fig. 3 F). The expression levels of Aire-driven genes were comparable to those of Aire-enhanced genes when the top 5% of high-expressing cells were compared (Fig. S2 F), indicating that the low frequency of cells expressing Aire-driven genes was not due to the “drop-out phenomenon” typically seen for genes showing low levels of expression (Kharchenko et al., 2014). Instead, expression from a limited fraction of cells represented the stochastic nature of Aire-driven genes. Thus, RamDA-seq analysis revealed distinct expression patterns for Aire-driven genes and Aire-enhanced genes, which were further validated in the following analysis.

### Distinct regulatory mechanisms for the expression of Aire-DEGs

The co-expression of a set of genes is a vital feature characterizing gene regulatory networks and can give us important insight into their mechanisms (Yin et al., 2021). We therefore computed intergene Pearson’s correlations of Aire-driven genes within Aire-expressing mTECs (RamDA-c2) and Aire-enhanced genes within other mTEC^high^ clusters (RamDA-c13, RamDA-c8, RamDA-c14, RamDA-c7, and RamDA-c11) to discern any unique regulatory patterns. We found that co-expression was frequent for the Aire-enhanced genes, forming discrete small clusters (micro-clusters) of co-expressed genes in the heatmap (Fig. 3 G, upper left, and Fig. S2 G). This suggests that Aire-enhanced genes are systematically co-expressed in the context of mTEC differentiation. In marked contrast, co-expression among the Aire-driven genes was remarkably rare (Fig. 3 G, lower left, and Fig. S2 G), suggesting their stochastic nature. When Aire-enhanced genes were co-expressed, they were mainly on the different chromosomes (93% interchromosomal) (Fig. 3 G, upper right). In case a rare co-expression was observed for the Aire-driven genes (Pearson’s correlation coefficient >0.6), their genomic positions were close on the same chromosomes (96% intra-chromosomal with a median distance of 20.4 kb) (Fig. 3 G, lower right, and Fig. 3 H, top). Furthermore, when Aire-driven genes shared the same chromosome, their locations were closer than those of the Aire-enhanced genes (Fig. 3 H, bottom). These results suggested that Aire-driven genes are coincidentally co-expressed due to their close genomic positions during the process of transcriptional activation by Aire. Of note, we rarely detected co-expressions when analyzing all Aire-DEGs within RamDA-c2 (Fig. S2, H and I), suggesting that both Aire-driven genes and Aire-enhanced genes are stochastically expressed in the Aire-expressing cells. Thus, our results demonstrated a distinct gene regulatory mechanism between Aire-expressing mTECs and other mTEC^high^ clusters for the production of Aire-DEGs as illustrated in Fig. 3 I. In Aire-expressing mTECs, Aire-DEGs are expressed as direct targets of Aire’s transcriptional activity without any coordinated expression patterns, as exemplified by Aire-driven genes. However, Aire-enhanced genes are also expressed in mimetic cells besides Aire-expressing mTECs. In this case, characteristic TFs that follow the developmental traits of peripheral tissues are employed to enable the coordinated expression of Aire-enhanced genes. Consistent with this idea, Gene Ontology (GO) analysis of Aire-driven genes showed no coherent features consistent with the promiscuous gene expression from mTECs, while Aire-enhanced genes in each cluster suggested their association with peripheral organs (Fig. S2 J).

**Figure 4.**
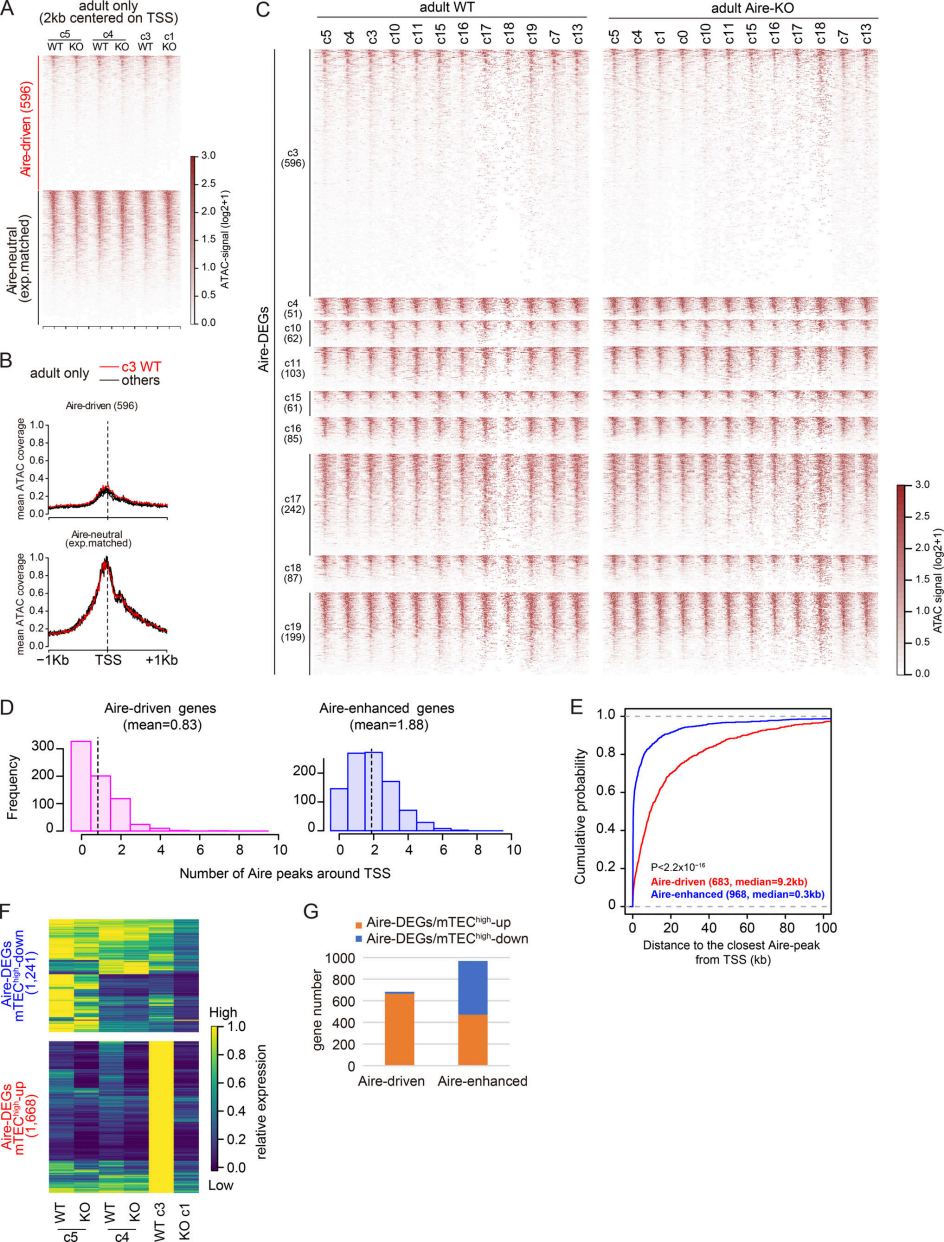
**Suppressive chromatin state in Aire-driven genes. (A)** Chromatin accessibility surrounding TSS, assessed by ATAC-seq in adult cells from the indicated clusters for Aire-driven genes and expression-matched Aire-neutral genes. **(B)** Mean chromatin accessibility traces surrounding TSS in adult WT cells in scMulti-seq/c3 (red) and other clusters (black) as indicated in Fig. 4 C. **(C)** ATAC signals surrounding TSS of Aire-DEGs up-regulated in each cluster, as indicated in Fig. 2 C, in adult WT cells (left) and adult Aire-KO cells (right) from the specified clusters. Genes with multiple TSS were excluded. **(D)** Histograms indicating the number of Aire peaks (q < 0.01) within a 20-kb window centered on TSS of Aire-driven genes (left) and Aire-enhanced genes (right). The mean number of peaks is 0.83 for Aire-driven genes and 1.88 for Aire-enhanced genes, with a statistical significance of P = 4.17 × 10^−63^ by the Wilcoxon rank-sum test. **(E)** CDF plot comparing the distance from TSS to the nearest Aire peak in Aire-driven and Aire-enhanced genes. The median distances are 9.2 and 0.3 kb for Aire-driven and Aire-enhanced genes, with a statistical significance of P < 2.2 × 10^−16^ by the Wilcoxon rank-sum test. **(F)** Expression heatmap of 2,909 Aire-DEGs shown in Fig. 2 A, color-coded relative to the maximum mean expression among the plotted clusters. **(G)** Bar graph indicating the number of Aire-DEGs/mTEC^high^-up and Aire-DEGs/mTEC^high^-down for two categories of Aire-DEGs (i.e., Aire-driven genes and Aire-enhanced genes). See Table S1 for a comprehensive list of these genes.

In order to dissect the chromatin configuration for Aire’s gene induction, we focused on Aire-driven genes and investigated the mean ATAC signals around their transcription start sites (TSS), i.e., accessibility of TSS. As a control, we selected genes that were not induced by Aire (Fig. S2 K; adult WT/c3 versus adult Aire-KO/c1, |log_2_FC| < 0.5; Aire-neutral genes). We found that Aire-driven genes had lower accessibility of TSS compared with Aire-neutral genes throughout the maturation from mTEC^low^ to mTEC^high^ in both WT and Aire-KO (c5→c4→c3 in WT; c5→c4→c1 in Aire-KO) (Fig. 4, A and B). In contrast, Aire-DEGs expressed in mTEC^high^ clusters outside c3 (i.e., Aire-enhanced genes from mimetic cells) showed higher accessibility in both WT and Aire-KO compared with Aire-driven genes (Fig. 4 C).

We further examined the state of Aire binding near Aire-driven genes by utilizing previously reported Aire chromatin immunoprecipitation followed by sequencing (ChIP-seq) data from bulk mTEC^high^ (Bansal et al., 2017). Of note, Aire-driven genes exhibited the lower number of Aire peaks around TSS (Fig. 4 D) and greater distance from TSS to the nearest Aire peak (Fig. 4 E) compared with Aire-enhanced genes. Thus, we confirmed distinct gene regulatory mechanisms for Aire-driven genes in Aire-expressing mTECs by their gene expression patterns (Fig. 3, F–H) and chromatin accessibility (Fig. 4, A–E).

### Mimetic cell genes constitute bulk Aire-induced genes

**Figure S3.**
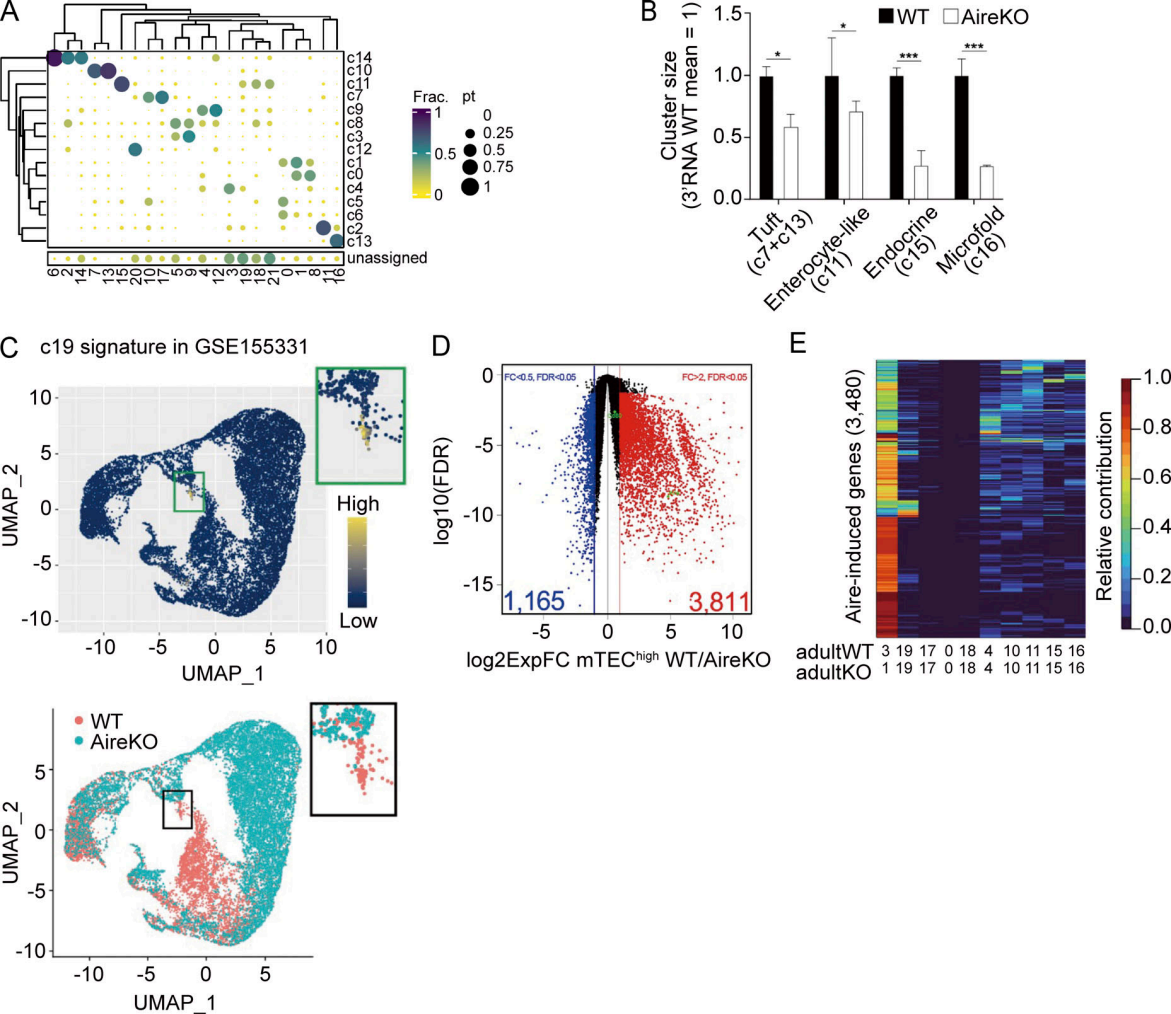
**Integrative analyses of scMulti-seq data with conventional scRNA-seq and bulk RNA-seq.** Related to Fig. 2. **(A)** Comparison of the clusters between scMulti-seq (query) and scRNA-seq (reference). The size and color of the circles represent the proportion of cells from the query cluster corresponding to the reference cluster. **(B)** Relative sizes of TEC subpopulations in adults calculated with the previously published scRNA-seq analysis. Population sizes were normalized by WT samples. P values by unpaired, two-sided Student’s *t* tests. **(C)** Corresponding cells for scMulti-seq-c19 in the published scRNA-seq dataset. UMAP visualizations of TECs color-coded according to the mean expression of 319 signature genes for scMulti-seq-c19 (top) and mouse genotypes (bottom). **(D)** Volcano plot comparing bulk RNA-seq of mTEC^high^ from WT (*n* = 7) and Aire-KO (*n* = 4). P values from quasi-likelihood F-tests were BH-corrected. Bulk Aire-induced genes (expression FC [ExpFC] > 2 and FDR < 0.05) are highlighted in red. **(E)** Analysis of the contribution of each cluster to decreased expressions of bulk Aire-induced genes in mTEC^high^. Corresponding clusters in WT and Aire-KO are indicated at the bottom. WT/c3 showed the highest contribution for 82.2% of genes.

Distinguishing Aire’s transcriptional targets and genes expressed by mimetic cells would help understand how mTECs mediate self-tolerance in the thymus. As mentioned above, a substantial portion of Aire-DEGs was expressed by several mTEC^high^ clusters, including mimetic cells, besides Aire-expressing mTECs. We also noticed that the sizes of some mimetic cell clusters were reduced in adult Aire-KO compared with adult WT (Table S1), and this observation was supported by another study (Michelson et al., 2022). To statistically evaluate the effect of Aire deficiency on the production of mimetic cell clusters in our cohort, we utilized our previous scRNA-seq analysis dataset (GSE155331) (Nishijima et al., 2022) as biological replicates. By cell-to-cluster matching based on gene expression signatures, we first identified matched clusters in the previous scRNA-seq data with the current scMulti-seq data (Fig. S3 A). We confirmed the reduced sizes of clusters representing thymic tuft cells (c7+c13), enterocyte-like mTECs (c11), endocrine mTECs (c15), and microfold mTECs (c16) from adult Aire-KO (Fig. S3 B). Ciliated cells (c19) were also diminished in Aire-KO by the present scMulti-seq data (Table S1), as well as the previous scRNA-seq data (Nishijima et al., 2022) (Fig. S3 C). This suggests that altered compositions in these mimetic cell clusters constitute the bulk Aire-induced genes. We then asked to what extent Aire-DEGs existing in each cluster contributed to the Aire-induced genes defined by the bulk mTEC^high^ analysis (Fig. S3 D and Table S3). Considering expression levels and the cluster size, we found that Aire-DEGs in WT/c3 most highly contributed to the Aire-induced genes among the mTEC^high^ clusters (Fig. S3 E). Additionally, genes expressed in mTEC^high^ clusters outside the WT/c3 variably contributed to the expression of Aire-induced genes. For example, reflecting the effect of significant size reduction, c19 (i.e., ciliated cells) highly contributed to a part of Aire-DEGs (Fig. S3 E). Thus, although bulk Aire-induced genes highly reflect Aire’s transcriptional activity in Aire-expressing cells, Aire-enhanced genes expressed outside the Aire-expressing cells (represented by mimetic cells) also contribute to the bulk Aire-induced genes.

### Expression dynamics of Aire-DEGs during mTEC maturation

We next validated the two distinct types of Aire-DEGs (i.e., Aire-driven genes and Aire-enhanced genes) by monitoring their expression dynamics during the maturation from mTEC^low^ to mTEC^high^. Among the 2,909 Aire-DEGs, 1,668 genes (57%) were up-regulated during the maturation (i.e., c5 → c4 → c3) in WT (Fig. 4 F, lower half). Unexpectedly, 1,241 genes (43%) were rather down-regulated during the maturation (Fig. 4 F, upper half). We called the former type of Aire-DEGs “Aire-DEGs/mTEC^high^-up,” whereas we called the latter type “Aire-DEGs/mTEC^high^-down.” In the adult Aire-KO, we did not see the up-regulation of Aire-DEGs/mTEC^high^-up during the maturation (i.e., c5 → c4 → c1), as expected (Fig. 4 F, lower half). In contrast, down-regulation of Aire-DEGs/mTEC^high^-down occurred similarly in Aire-KO as in WT (Fig. 4 F, upper half). We then asked which type of dynamic changes Aire-driven genes and Aire-enhanced genes manifest. We found that Aire-driven genes were almost exclusively Aire-DEGs/mTEC^high^-up (665 out of 683 genes), whereas Aire-enhanced genes were composed of both Aire-DEGs/mTEC^high^-up and mTEC^high^-down (Fig. 4 G). These results support our view that the expression of Aire-driven genes genuinely requires Aire’s transcriptional activity, and they are up-regulated upon Aire expression.

### PRC2-mediated gene suppression plays an important role in the induction of Aire-driven genes

**Figure 5.**
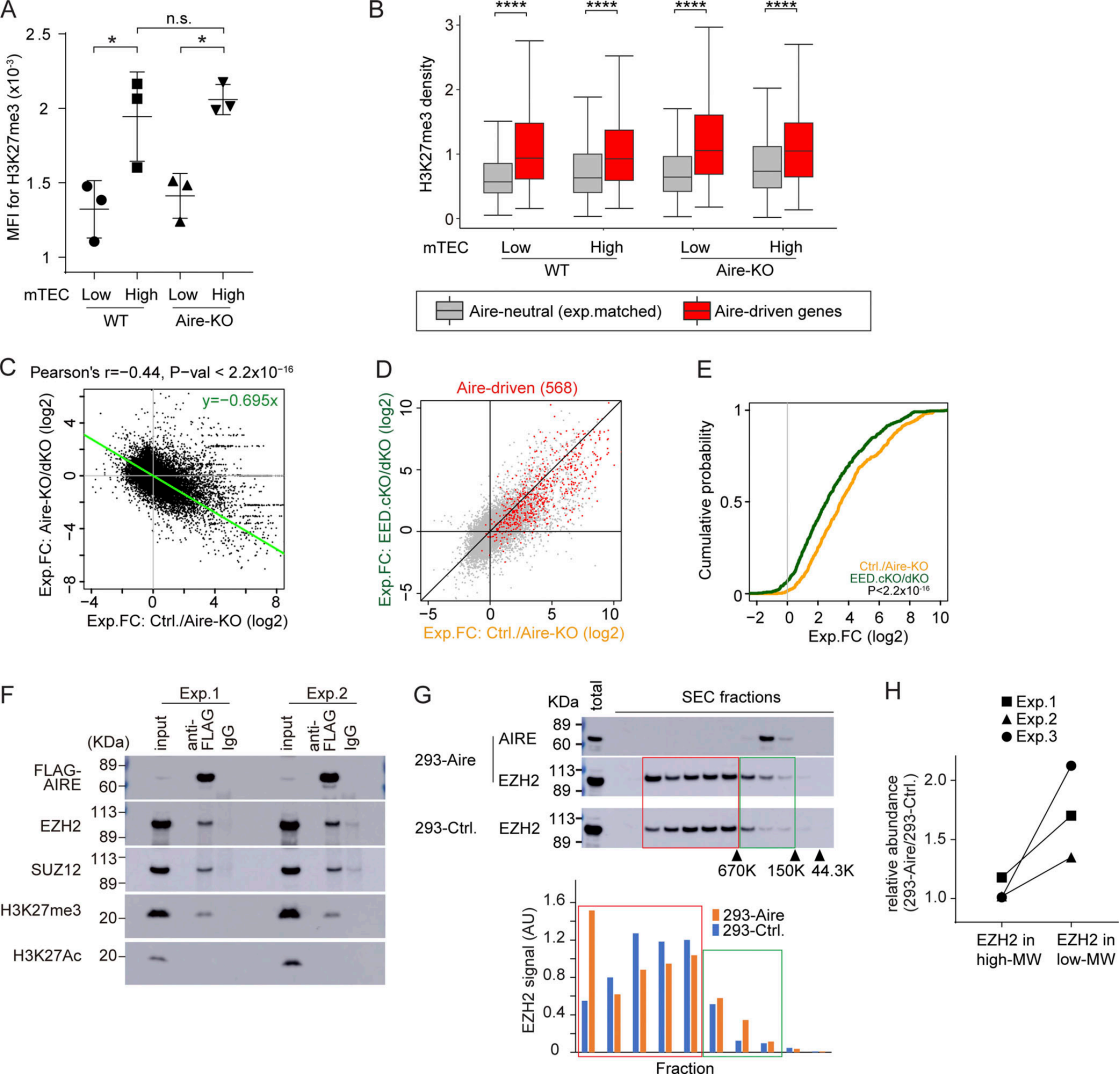
**Gene suppression by PRC2 is associated with gene induction by Aire. (A)** MFI of H3K27me3 in mTEC^low^ and mTEC^high^ from WT (*n* = 3) and Aire-KO (*n* = 3) by flow cytometric analysis. P values from one-way ANOVA followed by the Tukey–Kramer test. Representative flow plots are shown in Fig. S4 A. n.s., not significant; *, P < 0.05. **(B)** Box plots for mean H3K27me3 density in gene loci ± 10-kb regions of Aire-driven genes and Aire-neutral genes in mTEC^low^ and mTEC^high^ for WT and Aire-KO determined by CUT&Tag (*n* = 2 for each). P values from one-way ANOVA test followed by Tukey’s HSD method. ****, P < 0.0001. **(C)** FC-FC plot comparing gene regulations by Aire (x axis) and EED (y axis) in mTEC^high^ evaluated by bulk RNA-seq analysis (*n* = 3 for Aire-KO and Aire/EED-dKO, *n* = 5 for Ctrl.). The green trendline represents the SMA regression. Pearson’s correlation coefficient and P value by the F-test are indicated at the top. **(D)** FC-FC plot comparing Aire-mediated gene induction under EED-sufficient (x axis) and EED-deficient conditions (y axis) assessed by RNA-seq analysis (*n* = 3 for Aire-KO, EED-cKO, and Aire/EED-dKO, *n* = 5 for Ctrl.), with Aire-driven genes highlighted in red. **(E)** CDF plot comparing the up-regulation of Aire-driven genes by Aire in EED-sufficient and EED-deficient conditions (orange and green line, respectively), with a P value by the Wilcoxon signed-rank test. **(F)** Co-IP analysis of PRC2 and H3K27me3 interactions with Aire. Nuclear extracts from 293FT cells expressing Flag-tagged Aire were incubated with anti-Flag Ab or control IgG. Results from two independent experiments. **(G and H)** SEC profiles for EZH2 in 293FT cells transfected with Flag-tagged Aire or control plasmid. Arrowheads indicate the positions of standard markers. The relative abundance of EZH2 in PRC2 dimer (higher MW, red box) and monomer (lower MW, green box) is indicated. “Total” refers to the sample before SEC fractionation (see also Fig. S4 E). Results from three independent experiments are summarized in H. MFI, median fluorescence intensity. Source data are available for this figure: SourceData F5.

**Figure S4.**
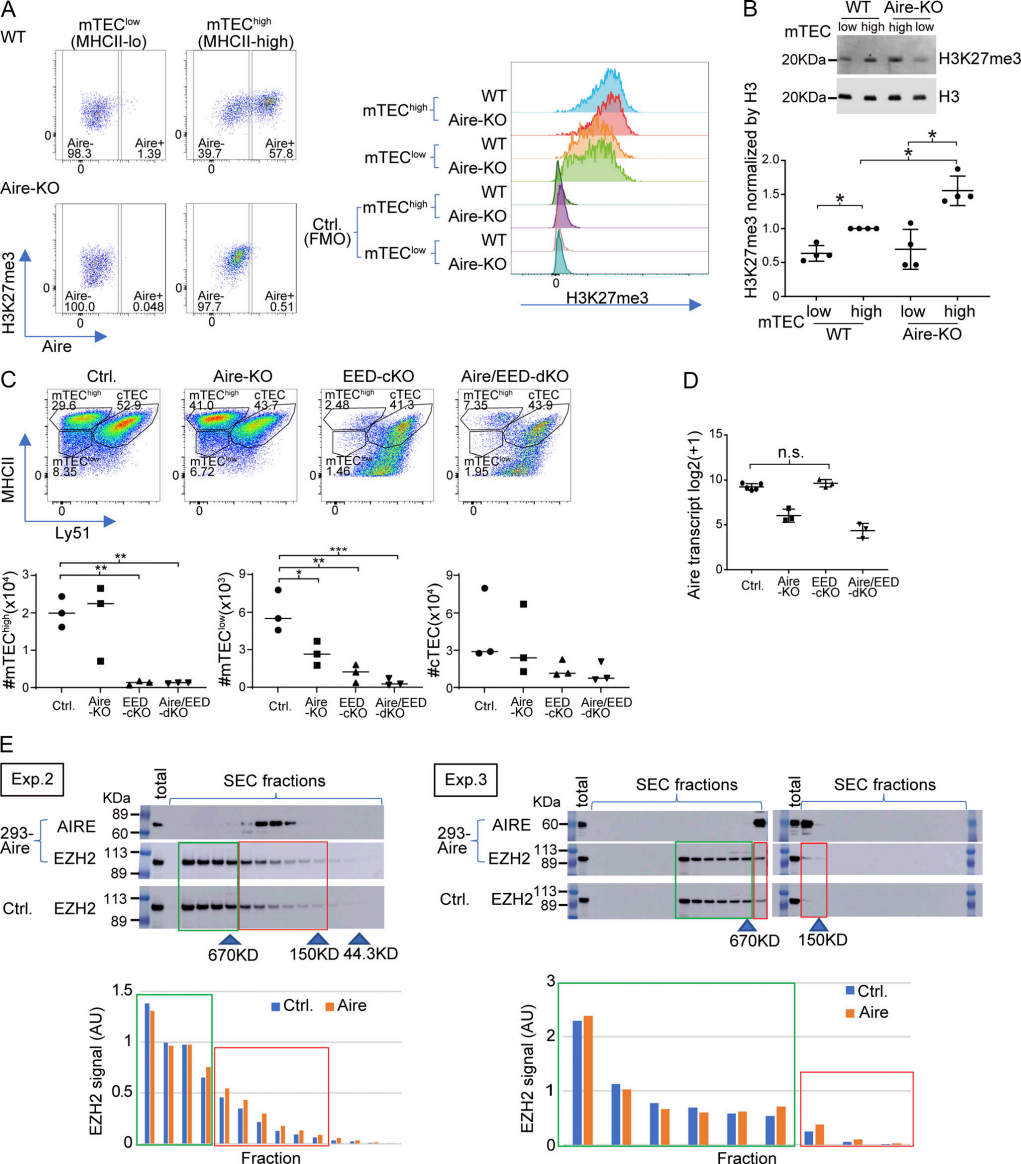
**Gene suppression by PRC2 is associated with gene induction by Aire.** Related to Fig. 5. **(A)** Left: Representative flow plots depicting Aire expression and H3K27me3 modifications in mTEC^low^ (left) and mTEC^high^ (right) from WT (top) and Aire-KO (bottom). Right: Histograms comparing H3K27me3 among the different populations. FMO served as a control. **(B)** Analysis of H3K27me3 in mTECs from WT and Aire-KO by western blotting. A typical western blot (top) and summarized quantification after normalization by the H3 signals (mean ± SD from four experiments), with P values by one-way ANOVA followed by the Tukey–Kramer test (bottom). **(C)** Top: Representative flow plots showing TEC subpopulations in control, Aire-KO, EED-cKO, and Aire/EED-dKO at 1- to 6-day-old, gated on the DAPI^−^CD45^−^EpCAM^+^ population. Bottom: Summary of the TEC subpopulation cellularity in each genotype (*n* = 3 for each). **(D)** Aire transcripts in mTEC^high^ evaluated by RNA-seq in indicated genotypes of mice. Each dot represents an individual mouse, with P values by one-way ANOVA followed by the Tukey–Kramer test. **(E)** SEC profiles for EZH2 in 293FT cells transfected with Flag-tagged Aire or control plasmid, analyzed by western blotting, as shown in Fig. 5 G. FMO, fluorescence minus one. Source data are available for this figure: SourceData FS4.

H3K27me3 is an epigenetic hallmark of the suppressive chromatin state. Although it has been previously reported that Aire-induced genes are marked by high levels of H3K27me3 (Sansom et al., 2014), the actual contribution of H3K27me3 in Aire’s gene induction has not been fully demonstrated. We first examined the H3K27me3 densities during the maturation using bulk mTECs. The mTEC^high^ fraction showed higher levels of H3K27me3 compared with mTEC^low^ by flow cytometric analysis (Fig. 5 A and Fig. S4 A) and western blot analysis (Fig. S4 B), suggesting the overall progressive chromatin suppression during the maturation. To specifically focus on the H3K27me3 status on Aire-driven genes, which represent Aire’s transcriptional activity, we employed Cleavage Under Targets and Tagmentation (CUT&Tag) with spike-ins for normalizations across mTEC^high^ and mTEC^low^ (Greulich et al., 2021). Irrespective of the maturation status (mTEC^low^ versus mTEC^high^) and Aire (WT versus Aire-KO), Aire-driven genes showed higher H3K27me3 densities compared with Aire-neutral genes (Fig. 5 B). Thus, Aire-driven genes are subjected to the chromatin suppression throughout the maturation, and Aire may overturn this for the gene induction. In this scenario, it is possible that chromatin suppression by H3K27me3 is a prerequisite for the induction of Aire-driven genes.

We tested this idea by focusing on the PRC2, which is solely responsible for the deposition of the H3K27me3 mark. Because embryonic ectoderm development (EED) is a core component of PRC2, we crossed EED-floxed mice (Yu et al., 2009) with Foxn1-Cre mice (Gordon et al., 2007) to generate PRC2-deficient TECs (EED-cKO). We also prepared mice deficient for both EED and Aire by crossing EED-cKO with Aire-KO (Aire/EED-dKO) and analyzed their transcriptome (Table S3). EED-cKO showed a significant reduction in mTEC^low^ and mTEC^high^ fractions compared with control mice (Fig. S4 C), which was consistent with the β5t-Cre–mediated deletion of EED-floxed allele in mice (Barthlott et al., 2021). Although the mTEC^high^ population was significantly decreased in EED-cKO, the expression level of Aire mRNA was not altered (Fig. S4 D). A decrease in mTEC^high^ fraction was also observed in Aire/EED-dKO (Fig. S4 C), indicating that PRC2 plays an important role in developing TECs besides gene regulation. Utilizing these models, we investigated the relationship between gene suppression by PRC2 and gene induction by Aire. We first evaluated Aire’s gene induction by calculating the fold change (FC) of gene expression levels between WT and Aire-KO in mTEC^high^ (Fig. 5 C; x axis). We then compared it with the effect of EED by calculating the FC between Aire-KO and Aire/EED-dKO (Fig. 5 C; y axis). Remarkably, we found an inverse correlation between the two. The results suggested that gene suppression by EED preconditions Aire targets to be effectively induced. To test this hypothesis, we evaluated the induction of Aire-driven genes by Aire, either in the presence of EED (Fig. 5 D; x axis) or in the absence of EED (Fig. 5 D; y axis). The effect of Aire’s gene induction was weaker when EED was not present, assessed by the FC-FC plot (Fig. 5 D) and cumulative distribution function (CDF) plot (Fig. 5 E). Our results suggested that chromatin suppression by EED (PRC2) contributes to Aire’s gene induction.

### Aire unleashes the silenced chromatin configuration caused by PRC2

To understand how Aire promotes gene expression by overcoming the suppressed chromatin marked with H3K27me3, we utilized 293 cells transfected with Aire cDNA, because the number of Aire-expressing mTEC is not sufficient to perform biochemical experiments (Abramson et al., 2010; Giraud et al., 2012; Yoshida et al., 2015). We found that PRC2 components (Ezh2 and Suz12) and H3K27me3 were coimmunoprecipitated with Aire, indicating their physical interaction (Fig. 5 F). The results suggest that Aire is preferentially recruited to the suppressed chromatin domain through its physical affinity. Because it has been suggested that PRC2 dimers are more effective in transcriptional suppression than PRC2 monomers (Grau et al., 2021), we examined the effect of Aire on the formation of the PRC2 dimer/monomer using size-exclusion chromatography (SEC) coupled with western blot analysis (Fig. 5 G and Fig. S4 E, and summarized in Fig. 5 H). The results indicated that Aire promotes the formation of smaller-sized PRC2 complexes compared with the mock-transfected cells, suggesting that Aire converts PRC2 dimers into a less suppressive form of monomers. Thus, our results propose a mechanism for Aire’s gene control: Aire is recruited to the suppressed chromatin site and destabilizes PRC2 dimers into monomers, thereby desuppressing the transcription of the targets.

### PRC2 controls the development of mimetic cells

**Figure S5.**
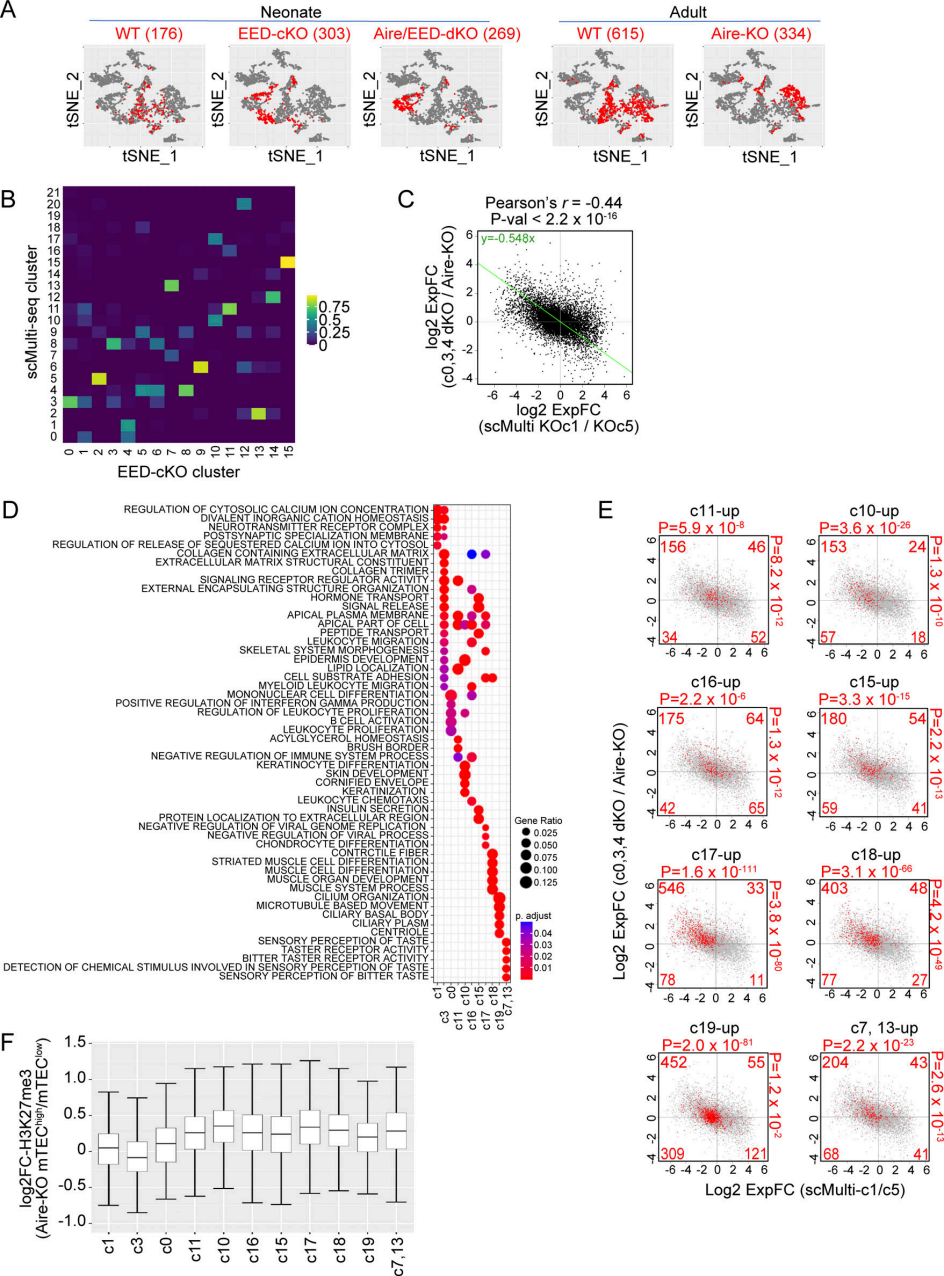
**PRC2 contributes to Aire-mediated gene expression and the development of mimetic cells.** Related to Fig. 6. **(A)** t-SNE visualization of 2,552 TECs. Cells sorted as mTEC^high^ (CD45^−^EpCAM^+^Ly51^−^MHC-II^high^) are color-coded according to mouse age and genotypes. Cell numbers for each group are indicated on the top. **(B)** Confusion matrix indicating the corresponding clusters for Fig. 6 A (query) in scMulti-seq (reference) shown in Fig. 1 A, with normalization for each column (query). **(C)** FC-FC plot comparing gene regulation during mTEC maturation (x axis) and PRC2-mediated gene regulation (y axis), with a green trendline representing the SMA regression. Pearson’s correlation coefficient and P value by the F-test are indicated at the top. **(D)** GO analysis of DEGs predominantly up-regulated in each cluster shown in Fig. 6 G. P values from the hypergeometric distribution were BH-corrected. **(E)** FC-FC plot comparing gene regulation during mTEC maturation (x axis) and PRC2-mediated gene regulation (y axis), with the mimetic cell genes from Fig. 6 G highlighted in red (see also Fig. 6 H). **(F)** Box plots indicating the difference in H3K27me3 density in mTEC^low^ and mTEC^high^ from Aire-KO for gene loci ± 10-kb regions of mimetic cell genes up-regulated in the indicated clusters (see also Fig. 6 G), determined by CUT&Tag (*n* = 2 for each).

**Figure 6.**
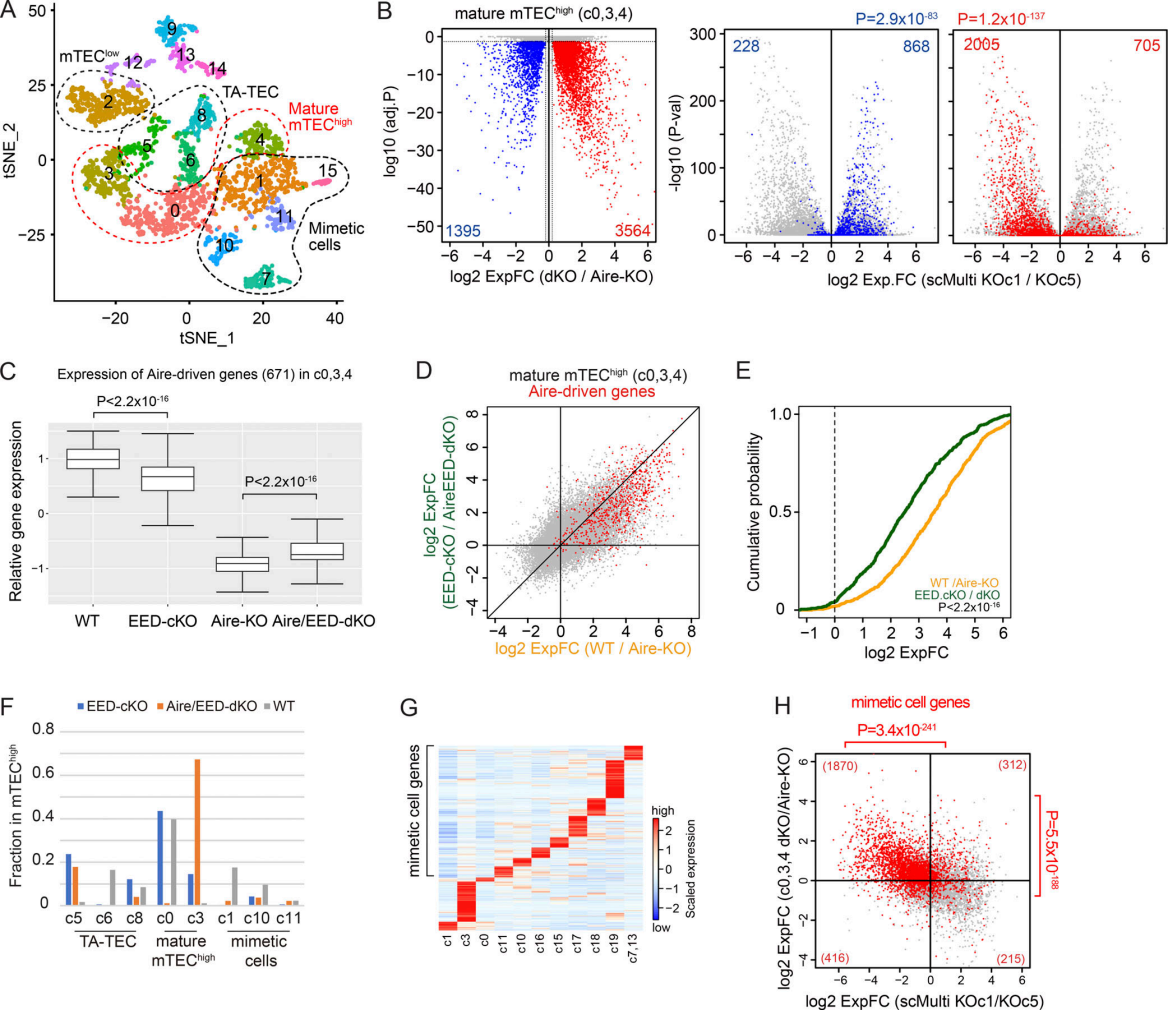
**PRC2 suppresses genes associated with mimetic cell populations. (A)** t-SNE visualization of 2,552 TECs from WT, Aire-KO, EED-cKO, and Aire/EED-dKO, color-coded by cluster assignment. mTEC^high^ from each group is indicated in Fig. S5 A. **(B)** Expression of PRC2-regulated genes. Left: Volcano plot comparing mature mTEC^high^ populations (c0, c3, and c4) from Aire/EED-dKO and Aire-KO, with the Bonferroni-adjusted P values from the Wilcoxon rank-sum test. Up-and-down DEGs (|log_2_ExpFC| > 0.2 & P.adj < 0.05) are highlighted in red and blue, respectively. Center and right: Volcano plot comparing mTEC^low^ and mTEC^high^ from Aire-KO (scMulti KO/c1 versus KO/c5), highlighted for DEGs in the left panel, with P values by the chi-square test against random distribution. **(C)** Box plot for the mean expression of Aire-driven genes (scaled log[GeTMM+1]) in indicated clusters. P values from one-way ANOVA test followed by Tukey’s HSD method. **(D)** FC-FC plot comparing Aire-mediated gene induction under EED-sufficient (x axis) and EED-deficient conditions (y axis) in mature mTEC^high^ clusters (c0, c3, and c4), with Aire-driven genes highlighted in red. **(E)** CDF plot comparing the up-regulation of Aire-driven genes by Aire in EED-sufficient and EED-deficient conditions (orange and green line, respectively), with a P value by the Wilcoxon signed-rank test. **(F)** Relative sizes of TEC subpopulations in cells sorted as mTEC^high^ (CD45^−^EpCAM^+^Ly51^−^MHC-II^high^) for each genotype. Small clusters, where the maximum size is <2%, were excluded. **(G)** Heatmap of 11,230 DEG (log_2_ExpFC > 1 & P.adj < 0.05) expression across mature mTEC^high^ and mimetic cell clusters, represented by standardized expressions among the plotted clusters. Genes were grouped according to their expression patterns. Genes up-regulated in c11 to c7,13 were grouped as mimetic cell genes. **(H)** FC-FC plot comparing gene regulation during mTEC maturation (x axis) and PRC2-mediated gene regulation (y axis), with the mimetic cell genes highlighted in red (see also Fig. S5 E).

The above results demonstrated that PRC2 plays an important role in the induction of Aire-driven genes. However, considering the altered composition of TECs by the lack of EED (Fig. S4 C), compositions in mTEC^high^ may also contribute to the altered transcriptome in bulk mTEC^high^, similar to what we have seen in mTEC^high^ from Aire-KO (Nishijima et al., 2022). Therefore, we have prepared another set of RamDA-seq data using mTEC^high^ in neonate WT, EED-cKO, and Aire/EED-dKO. Then, we integrated this cohort with the previous RamDA-seq dataset (Fig. 3 A), comprising total TECs from adult WT and Aire-KO (Fig. S5 A). We identified a total of 16 clusters (Fig. 6 A), including three clusters (c0, c3, and c4) corresponding to Aire-expressing or Aire-less mTECs based on the cell-to-cluster matching analysis using scMulti-seq clusters (Fig. S5 B; mature mTEC^high^ hereafter). Mature mTEC^high^ in each sample shaped different clusters depending on their genotypes (Fig. S5 A). Mimetic cell clusters (c1, c7, c10, c11, and c15) were also identified among the mTEC^high^ fraction (Fig. 6 A and Fig. S5 A).

Knowing that PRC2 regulates mTEC heterogeneity, we next examined the effect of the lack of PRC2 on the transcriptome by focusing on the mature mTEC^high^ clusters. When the expression profile was compared between Aire/EED-dKO and Aire-KO, 3,564 genes were up-regulated, while 1,395 genes were down-regulated (Fig. 6 B, left). Up-regulated genes and down-regulated genes in the mature mTEC^high^ by the loss of EED were consistent with the transcriptomic changes during mTEC^low^ (Aire-KO/c5) to mTEC^high^ (Aire-KO/c1) in scMulti-seq clusters from Aire-KO (Fig. 6 B, middle and right). Furthermore, the FC-FC plot comparing the mTEC maturation process (x axis: scMulti-seq Aire-KO/c5 versus Aire-KO/c1) and the gene suppression by PRC2 (y axis: RamDA-seq Aire/EED-dKO versus Aire-KO) exhibited a negative correlation (Fig. S5 C). The results suggested that PRC2 contributes to the transcriptomic changes from mTEC^low^ to mTEC^high^. When we compared the transcriptome within mature mTEC^high^ in each genotype, the defect of EED ameliorated the down-regulation of Aire-driven genes on Aire-KO background (Fig. 6 C; Aire/EED-dKO versus Aire-KO), whereas the defect of EED rather reduced the expression of Aire-driven genes on WT background (Fig. 6 C; EED-cKO versus WT). Indeed, consistent with the bulk analysis, the induction of Aire-driven genes by Aire was more profound when EED was present in mature mTEC^high^ (compare Fig. 6, D and E [RamDA-seq] with Fig. 5, D and E [bulk RNA-seq]). The results further supported our idea that PRC2 mediates gene suppression from mTEC^low^ to mTEC^high^ irrespective of Aire, while the coexistence of Aire overrides the chromatin suppression by PRC2 to induce the targets.

When the relative population size of the cells in each RamDA-seq cluster was examined, we found that both EED-cKO and Aire/EED-dKO showed a significant reduction of mimetic cell cluster size, which was more apparent than the effect caused by Aire deficiency (Fig. 6 F). We then examined the expression dynamics of genes related to mimetic cells during mTEC maturation. We selected a set of genes representing the mimetic cells by searching the genes predominantly expressed in each scMulti-seq mTEC^high^ cluster (Fig. 6 G). GO analysis of these genes indicated their association with peripheral organs, as expected (Fig. S5 D), and we call them mimetic cell genes. We then overlaid these mimetic cell genes on Fig. S5 C to see their regulation during mTEC maturation (x axis: mTEC^high^ versus mTEC^low^) and their suppression by EED (y axis: Aire/EED-dKO versus Aire-KO) (Fig. 6 H). Interestingly, the expression of mimetic cell genes was significantly down-regulated during the mTEC maturation (x axis in Fig. 6 H and Fig. S5 E), while they were significantly up-regulated if the activity of PRC2 was eliminated (y axis in Fig. 6 H and Fig. S5 E). This indicated that mimetic cell genes were suppressed by PRC2 during the mTEC maturation. Consistent with the function of PRC2, H3K27me3 modification of the mimetic cell genes in mTEC^high^ was higher than that in mTEC^low^ when assessed by a CUT&Tag method (Fig. S5 F). Taken together, our results demonstrated that PRC2 is responsible for the physiological gene suppression during mTEC maturation for the expression of Aire-driven genes together with the development of mimetic cells.

## Discussion

By revealing the characteristics of each mTEC cluster using scMulti-seq analysis, we have identified two distinct types of Aire’s gene control: genes expressed only in the Aire-expressing cells (i.e., Aire-driven genes) and those also induced in other mTEC^high^ clusters (i.e., Aire-enhanced genes). We validated these two distinct types of Aire’s gene induction in several ways: expression dynamics during mTEC maturation, GO analysis, and the expression pattern in individual cells using RamDA-seq. Analysis of gene expression during mTEC maturation, as presented in Fig. 4, F and G, revealed that approximately half of the Aire-enhanced genes exhibited reduced expression in Aire-expressing mTEC^high^ (WT/c3) compared with mTEC^low^ (WT/c5), where Aire is absent. Given that gene expression levels are normalized to the total RNA count, a decrease in expression does not necessarily indicate a reduction in the absolute abundance of RNA per cell. However, since genes induced by Aire generally exhibited an inverse correlation with those suppressed by PRC2 (Fig. 5 C), these findings strongly suggest that PRC2-mediated gene repression contributes to gene induction by Aire, emphasizing the importance of examining gene regulation by Aire within the broader context of transcriptional regulation during mTEC maturation. Furthermore, significant differences exist between Aire-driven and Aire-enhanced genes, as demonstrated in the present study and further discussed below. Thus, classifying Aire-DEGs into these two categories provides valuable insights for downstream analyses to elucidate the Aire-mediated gene control.

When we evaluated the expression of Aire-DEGs across mTEC^high^ clusters by scMulti-seq, 683 genes out of 2,909 Aire-DEGs were solely regulated in an Aire-driven manner. Using bulk mTEC^high^, Sansom et al. reported that 594 out of 3,980 Aire-induced genes were entirely dependent on Aire, whereas the expression levels of the rest were augmented by Aire (Sansom et al., 2014). Although Sansom’s study did not approach the molecular basis for the two distinct modes of gene regulation by Aire, our study and theirs demonstrated that the numbers of Aire’s transcriptional targets unique to Aire-expressing mTECs are rather limited. However, because of the absolute requirement of Aire for the expression of Aire-driven genes, they may have a chance to become the targets of autoimmune attacks in Aire-KO.

In contrast to Aire-driven genes, Aire-enhanced genes were also expressed in the mimetic cell clusters even from Aire-KO, furnishing unique transcriptional features to each cell type. While Aire-enhanced genes showed stochastic expression only in the RamDA-c2 (corresponding to scMulti-c3) (Fig. S2, H and I), how the expression of Aire-enhanced genes is differentially controlled in Aire-expressing cluster and mimetic cell clusters remains unknown. Investigating the elements such as enhancers that act on Aire-DEGs in cis and/or trans at a single-cell level might give us an important clue for the underlying stochastic mechanisms (Kim and Wysocka, 2023; Chowdhary and Benoist, 2023).

Self-reactive T cells in the thymus are either eliminated from the repertoire or develop into Tregs, depending on the avidity between TCRs and self-antigens. However, it has been reported that the geographic pattern of peptide presentation on individual cells may also play a role in the fate decision of self-reactive T cells (Banerjee and Chapman, 2018). Bioinformatics approaches have suggested that diverse, low-abundant self-peptide presentation would favor Treg differentiation (Khailaie et al., 2014). Because the response of T cells may depend on the complete set of interaction energies rather than on any single interaction exceeding a threshold, stochastic antigen presentation by Aire-expressing mTECs may play an important role in the development of Tregs. Consistent with this scenario, Aire has been suggested to enforce immune tolerance by modulating autoreactive T cells to differentiate into the Treg cell lineage (Malchow et al., 2016).

Because Aire-driven genes showed no obvious biological relevance, their stochastic expression pattern may hold immunological significance, as discussed above. Accordingly, it would be important to know not only the repertoire of self-antigens but also how the expression pattern of self-antigens is determined with the help of Aire. Some studies have emphasized the stochasticity of TRA gene expression (Derbinski et al., 2008; Villaseñor et al., 2008), whereas others have concluded the existence of a coordinated expression pattern for TRAs (Brennecke et al., 2015; Meredith et al., 2015; Baran-Gale et al., 2020). In this regard, our present study provides a reasonable explanation for the coregulation of TRA genes: stochastic expression of Aire-driven genes and coordinated expression of Aire-enhanced genes, the latter is most likely associated with the Aire-mediated mTEC development. This conclusion was certainly obtained by revisiting the functionality of Aire at the single-cell level by integrating high-sensitive single-cell approaches (Matsumoto et al., 2023). The coexistence of these two types of genes (i.e., stochastic expression of Aire-driven genes and coordinated expression of Aire-enhanced genes) would contribute to the complicated expression patterns in mTEC^high^ fraction in bulk RNA-seq, which have been referred as “ordered stochasticity” (Meredith et al., 2015).

Because it has been considered that mTECs acquire the ability to express a wide variety of TRAs as they mature, it was somewhat unexpected that Aire-DEGs were rather suppressed during maturation. Indeed, Aire-DEG/mTEC^high^-down showed a gross down-regulation from mTEC^low^ to mTEC^high^ at cluster levels (Fig. 4 F). However, this trend was due to a limited fraction of mTEC^high^ expressing high levels of Aire-DEGs, which was obscured when calculating the average expression for each gene by clusters. Our findings underscore that focusing solely on genes up-regulated from mTEC^low^ to mTEC^high^ may overlook a critical dimension of Aire’s gene regulatory mechanisms. This down-regulation was associated with the enhanced repressive chromatin mark H3K27me3 and regulated by PRC2. Although the enhancement of H3K27me3 in Aire-induced genes was previously suggested (Sansom et al., 2014), the exact mechanisms controlling the interaction between the repressive chromatin H3K27me3 and Aire remained unknown. We speculate that Aire is recruited to the repressive chromatin mark through the affinity between Aire, H3K27me3, and PRC2. In this regard, there is another report suggesting that Aire is recruited to the repressive sites formed by the ATF7ip-MBD1 complex (Waterfield et al., 2014). According to this scenario, Aire is considered to recognize the specific methylated CpG dinucleotides provided by MBD1 to target the loci of TRAs. In either case, gene suppression seems to be a prerequisite for the subsequent gene induction by Aire. The exact identification of the components of the Aire/PRC2 complex might be required to fully reveal the process of PRC2-mediated gene suppression.

In contrast to our findings, Barthlot et al. asserted that marking with H3K27me3 was not relevant to the Aire-mediated transcription of the targets using the EED^fl/fl^::β5t-Cre mouse model (Barthlott et al., 2021). However, it may be important to mention that their conclusion was based on the conventional scRNA-seq data showing no major difference in the expression levels of Aire-induced genes between EED-proficient and EED-deficient mTECs. In this regard, our highly sensitive RamDA-seq approach has enabled us to capture the down-regulation of Aire-driven genes in mTECs from EED^fl/fl^::Foxn1-Cre mice. Our results from Aire/EED-dKO, showing that the lack of EED rescued the transcriptional defect in Aire-KO mTECs, further supported our conclusion. Thus, we suggest Aire overrides PRC2-mediated chromatin suppression to induce its targets, while other factors preconditioning the Aire targets other than PRC2 remain to be studied. Considering the moderate perturbation of Aire-driven genes in EED-cKO compared with Aire-KO (Fig. 6 C), additional suppressive mechanisms, such as H3K4me0 (Org et al., 2008), may also play a role in Aire’s gene induction. However, our results certainly demonstrate that PRC2 is one of the key mechanisms in Aire-mediated gene regulation, and this mechanism makes biological sense considering that tissue-specific genes are expected to be silenced outside their tissue of origin in general.

We have demonstrated that Aire unleashes the repressive chromatin of the target loci by establishing a less suppressive chromatin form of PRC2 to induce the expression of Aire targets. However, the role of PRC2 in controlling the transcriptome from mTECs was not confined to this Aire-mediated gene expression. Instead, we found that PRC2 is also involved in the organization of mimetic cells: our single-cell analysis using mTECs deficient in both EED and Aire has demonstrated that the loss of PRC2 resulted in changes in the expression of genes unique to the mimetic cells together with the reduction of their population size. The results suggested that mimetic cell genes are subjected to gene suppression by PRC2 during mTEC maturation, thereby shaping each mimetic cell cluster. Furthermore, this action of controlling the development of mimetic cells was more apparent than Aire. Given that Aire and PRC2 independently organize the development of mimetic cells besides the cooperative induction of Aire-DEGs within the primary Aire-expressing mTECs, chromatin repression by PRC2 plays a much broader role in mTECs than previously thought.

Because of PRC2’s pleiotropic role, the exact role of PRC2 in controlling Aire-mediated gene expression may not be easy to reveal by separating from its developmental effect. However, our deep single-cell analyses and the biochemical approach have strongly suggested a link between Aire and PRC2 for chromatin configuration to control the Aire-mediated gene expression. Further studies are required to unveil how the Aire/PRC2 complex is recruited to the selected set of genes (Aire-driven genes) and particular genomic sites of the corresponding genes for their regulation.

Revisiting the published Aire ChIP-seq data (Bansal et al., 2017) to examine Aire peaks in proximity to Aire-driven and Aire-enhanced genes, we found distinct patterns; Aire peaks were located further from Aire-driven genes, whereas they were detected closer to Aire-enhanced genes (Fig. 4, D and E). This observation suggests that Aire may regulate Aire-driven and Aire-enhanced genes through different mechanisms, potentially acting on enhancers for Aire-driven genes and binding directly to promoter regions for Aire-enhanced genes. Alternatively, given that Aire-driven genes exhibit stochastic expression in a small subset of scMulti-c3, Aire localization at the promoter regions may be obscured when signals are averaged across the broader mTEC^high^ population in “bulk” ChIP-seq data. Moreover, since Aire-enhanced genes exhibit more uniform expression, consistent with classical regulatory mechanisms, in clusters other than scMulti-c3, the observed Aire binding could represent signals from those clusters other than scMulti-c3. We consider that bulk mTEC^high^ ChIP-seq data are inadequate to accurately assess Aire’s stochastic binding, and the stochastic nature of the Aire-mediated gene control would become evident only by the ChIP-seq at a single-cell resolution.

Finally, although we have focused on genes positively regulated by Aire (i.e., Aire-DEGs) in this study, there are also Aire-suppressed genes. CTLA-4 is one such gene, and we have reported the significance of ectopically expressed CTLA-4 from Aire-deficient mTECs in the breakdown of self-tolerance (Morimoto et al., 2022). Because Aire controls a wide variety of genes both positively and negatively, further studies are required for a complete understanding of the molecular action of Aire at play in multiple processes of tolerance induction.

## Materials and methods

### Mice

Aire-deficient mice on a C57BL/6 background have been reported previously (Yano et al., 2008). EED-flox mice (Yu et al., 2009) and Foxn1-Cre mice (Elderkin et al., 2007) were purchased from the Jackson Laboratory. The mice were maintained under pathogen-free conditions in the animal facility at the RIKEN Center for Integrative Medical Sciences. All animal experiments were performed in accordance with the protocol approved by Institutional Animal Care and Use Committees of RIKEN Yokohama Branch and with the animal care guidelines in RIKEN, Center for Integrative Medical Sciences. Unless otherwise specified, mice aged between 4 and 8 wk were utilized for the experiments.

### TEC isolation, flow cytofluorometrical analysis, and sorting

Preparation of thymic epithelial cells (TECs) and flow cytometric analysis was performed essentially as described previously (Nishijima et al., 2018). Specifically, thymi were dissected, and fat and connective tissues were trimmed off. Then, thymi were minced with a sharp pair of scissors in ice-cold RPMI-1640 medium without phenol red (Wako). The resulting thymic fragments were incubated in a digestion medium (RPMI-1640 medium without phenol red containing 0.5 mg/ml Liberase TH and 0.1 mg/ml DNase I; Roche) at 37°C for 15 min with mild agitation using ThermoMixer (Eppendorf). Cells were then further agitated by pipetting up and down using a P1000 tip and incubated for another 5 min at 37°C with agitation. Then, cells were agitated again using a P1000 tip until a single-cell suspension was obtained and filtered through 100-μm nylon mesh. After washing with MACS buffer (PBS containing 2 mM EDTA, 0.5% BSA, and 0.1% [wt/vol] NaN3), cells were resuspended in MACS buffer and incubated with CD45 microbeads (mouse CD45 microbeads, Miltenyi Biotec) at 4°C for 5 min. Then, a cocktail of antibodies (Abs) ([anti-]MHC class II [I-A–I-E] [M5/114.15.2], Ly51 [6C3], CD45 [30-F11], and EpCAM [CD326, G8.8]; all from BioLegend) was added and incubated at 4°C for 15 min. Cells were washed twice with MACS buffer and applied onto an LS column (Miltenyi Biotec) in the magnetic field to collect the flow-through enriched for CD45-negative cells. 4′,6-Diamidino-2-phenylindole dihydrochloride solution (DAPI) (Dojindo) was added to stain dead cells at 1 μg/ml. Cell analysis was performed using FACS CantoII and AriaIII instruments (BD). Cell sorting was performed using FACSAriaIII (BD) instrument. For analyzing H3K27me3, we used Zombie NIR Fixable Viability Kit (BioLegend), anti-H3K27me3 (C36B11; CST), DyLight405-conjugated anti-rabbit IgG (711-475-152; Jackson ImmunoResearch Laboratories), and eFluor660-conjugated anti-Aire (5H12; eBioscience) with Foxp3/Transcription Factor Staining Buffer Set (eBioscience). Data were analyzed using FlowJo software (BD).

### scMulti-seq library preparation

Cells were processed following the 10x Genomics protocol using Chromium Next GEM Single Cell Multiome ATAC + Gene Expression Reagent (10xGenomics). Specifically, ∼40,000 FACS-sorted mouse TECs (DAPI^−^CD45^−^EpCAM^+^) from Aire knockout mice and WT mice (adult WT; a pool of one male and one female mouse at 5 wk of age, adult Aire-KO; one male at 5 wk of age, neonate WT and Aire-KO; a pool of three mice at 2–4 days old) were incubated in 0.1× lysis buffer (0.01% Tween-20, 0.01% NP-40, 0.001% digitonin, 10 mM Tris-HCl, pH 7.4, 10 mM NaCl, 3 mM MgCl_2_, 1% BSA, 1 mM dithiothreitol [DTT], 1 U/μl RNasin Plus Ribonuclease Inhibitor; Promega) for 3 min on ice to isolate nuclei. Nuclei were washed and resuspended in chilled Diluted Nuclei Buffer (1× Nuclei Buffer; 10x Genomics, 1 mM DTT, and 1 U/μl RNasin Plus Ribonuclease Inhibitor; Promega) to target 8,000 recovered nuclei per sample. Nuclei were processed following the 10x Genomics protocol, and the pooled libraries were sequenced in multiplex on the Illumina NextSeq 2000 platform using P3 Reagent Kit.

### scMulti-seq data processing

FASTQ files were processed using Cell Ranger ARC v2.0.0 software with the reference data (refdata-cellranger-arc-mm10-2020-A-2.0.0; 10x Genomics). Seurat (v4.0.4) (Hao et al., 2021) and Signac (v1.7.0) (Stuart et al., 2021) on R programming language (https://www.R-project.org/) were principally used to analyze the data. The dataset was processed through the following steps: first, we selected cells for calling ATAC-seq peaks, which met the criteria of 2,000–35,000 nCount_RNA (the total number of RNA molecules detected within a cell), 8,000–200,000 nCount_ATAC (the total number of unique ATAC-seq reads on tentative ATAC-seq peaks determined by Cell Ranger ARC for each sample), and a percent.mt (the percentage of mitochondrial genes in nCount_RNA) below 20%. Next, we used macs2 (v2.2.7.1) (Zhang et al., 2008) to call ATAC-seq peaks for each group, which were combined to generate a list of 244,410 peaks. We then filtered out peaks overlapping with blacklisted genomic regions and chrM homologous regions to eliminate potential artifacts (Amemiya et al., 2019; Yoshida et al., 2019), resulting in 242,648 peaks used for the following analysis. We used cells meeting the following criteria for subsequent analysis to exclude low-quality cells: nCount_ATAC; 4,000–100,000, nCount_RNA; 2,000–35,000, and percent.mt <20%. We eventually pooled 21,986 cells (5,980 adult WT cells, 8,531 adult Aire-KO cells, 3,704 neonate WT cells, and 3,771 neonate Aire-KO cells; see Table S1) and determined 22 clusters using WNN analysis employing Seurat v4, which enabled us to define cellular status based on multiple data types (Hao et al., 2021). To calculate normalized gene expressions in individual cells, feature counts based on UMI for each cell were divided by the total counts for that cell and multiplied by 10,000, and then natural-log–transformed using log (+1). The differentially expressed genes for each cluster were identified by the fast Wilcoxon rank-sum test utilizing the R package Presto (https://github.com/immunogenomics/presto). Motif activities representing deviations in chromatin accessibility across regions annotated with each TF binding motif in individual cells were computed using the 242,648 peaks mentioned above and the RunChromVAR function in Signac (v1.3.0) (Schep et al., 2017; Stuart et al., 2021), along with the motif database JASPAR2020 (Fornes et al., 2020). Over- and under-represented TF motifs in each cluster were determined using the fast Wilcoxon rank-sum test utilizing the R package Presto. To elucidate differential TF activities between WT and Aire-KO cells, characteristic TF motifs for each cluster were determined based on area under the ROC curve (auROC) (AUC > 0.75, computed using the R package Presto) among TFs showing high correlations between transcript amount and TF activity (|Pearson’s r| > 0.4). The differences between WT and Aire-KO cells were computed employing Student’s *t* test, and the resulting P values were corrected using the Bonferroni method. The frequency of Tn5 integration around TSS was computed using unique ATAC-seq fragments captured by the assay and normalized by a per-group scaling factor, which was calculated by multiplying the number of cells in the group by the mean sequencing depth for that group of cells. The CoveragePlot function in Signac was used to generate pseudobulk accessibility tracks.

### scMulti-seq data analysis and visualization

The Pearson correlation coefficient between RNA abundance and the corresponding motif activity of a TF was computed using the mean expression and mean motif score for each cluster. We performed permutation tests to calculate the statistical significance of the correlations, by shuffling either the cluster labels or the TF labels (100 permutations), and P values were calculated using a Z-test comparing the observed and the permuted correlation coefficient between TF expression and motif score. We employed the highest values (the least significant values) from the two permutation approaches to represent the significance of the correlation (Table S1). The trajectory and pseudotime analysis were performed using the R package Monocle 3 (Cao et al., 2019; Trapnell et al., 2014; Qiu et al., 2017). We set c5 (mTEC^low^) as the root cells for adults because mTEC maturation proceeds from mTEC^low^ to mTEC^high^ (Gray et al., 2007) and c12 for neonates considering their active cell cycle presumably reflecting the highest capacity as progenitor cells (Fig. S1 G). The cell cycle phases were computed using the CellCycleScoring function in Seurat with cell cycle genes reported by Tirosh et al. (2016). Normalized gene expressions were standardized by scaling to evaluate the expression of a set of genes in the individual cell. To identify genes predominantly associated with specific clusters, we assessed the differential gene expression in each cluster relative to other cells by employing the fast Wilcoxon rank-sum test, utilizing the R package Presto. A gene was attributed to a cluster if its expression was significantly higher (adjusted P value <1 × 10^−5^) exclusively in the cluster exhibiting the highest mean expression or if it was considerably higher than the expression in the second-highest cluster (expression Z-score [highest] – Z-score [second highest] >1); We categorized the genes up-regulated in scMulti-seq c3 as Aire-driven genes, and those up-regulated in other clusters as Aire-enhanced genes, based on their expression patterns shown in Fig. 2 C. In the absence of clusters demonstrating significantly high expression, a gene was considered up-regulated in multiple clusters with relatively higher expressions (expression Z-score >0). When multiple clusters showed significantly high expression (adjusted P value <1 × 10^−5^) but did not meet the criteria for assignment to a specific cluster, a gene was regarded as up-regulated across clusters whose expressions were not lower than those of clusters with significantly high expression (adjusted P value <1 × 10^−5^). Differential gene expressions between two groups of cells were analyzed using a Wilcoxon rank-sum test employing the R package Seurat. For comparing multiple cell groups, mean gene expressions for each cell group were computed on a logarithmic scale (Table S1) and standardized across cell groups to assess the genes of interest uniformly. One-way ANOVA, followed by Tukey’s test, was performed for pairwise comparisons among the cell groups. To evaluate the contribution of mTEC^high^ clusters to the bulk Aire-induced genes, we focused on 3,480 bulk Aire-induced genes that were down-regulated (logFC < 0) in adult Aire-KO mice compared with WT within a pseudobulk population corresponding to mTEC^high^ (i.e., c0, c1, c3, c4, c10, c11, c15–19). We evaluated the expression from a cluster as the product of its mean expression and relative size in mTEC^high^.

### Analysis of TRA genes

We determined 7,764 TRA genes (Table S1) as those expressed in three organs or fewer by employing the publicly available gene expression database on BioGPS (Wu et al., 2009).

### GO analysis

GO enrichment analysis of a gene set and gene set enrichment analysis were performed using R with packages GO.db (3.15.0) (Carlson, 2022), clusterProfiler (4.4.4) (Wu et al., 2021), and msigdbr (Dolgalev, 2022). The enrichment P value calculation (i.e., the number of genes in the list that hit a given biology class compared with a purely random chance) was performed using the Benjamini–Hochberg multiple testing correction.

### RamDA-seq library preparation

RamDA-seq libraries were prepared following the previously described method (Hayashi et al., 2018). In brief, mTEC^high^ (DAPI^−^CD45^−^EpCAM^+^Ly51^−^MHC-II^high^), mTEC^low^ (DAPI^−^CD45^−^EpCAM^+^Ly51^−^MHC-II^low^), and cTEC (DAPI^−^CD45^−^EpCAM^+^Ly51^+^) were sorted using FACSAriaIII (BD) into a 96-well plate containing 1 μl of single-cell lysis buffer (1/10 10X lysis buffer; Takara, with 0.3% NP-40; Thermo Fisher Scientific, 1 U/μl RNasin Plus; Promega). RNA was denatured by incubating at 70°C for 90 s, followed by DNase I treatment (0.1 U/μl DNase I, Amplification Grade; Invitrogen) in 0.25× RT buffer (PrimeScript RT Reagent Kit; Takara) at 30°C for 5 min. Then, cDNA was synthesized in 3 μl of 1× RT buffer containing RT Enzyme Mix (0.15 μl of PrimeScript RT Enzyme Mix I; Takara), oligo(dT)-RT primer (0.2 μM Oligo[dT]18 Primer; Thermo Fisher Scientific), T4 gene 32 protein (0.033 mg/ml; NEB), and first-NSR primer for mouse (2.7 μM; SIGMA custom DNA oligos), by incubating for 10 min at 25°C, 10 min at 30°C, 30 min at 37°C, 5 min at 50°C, and 5 min at 94°C. Subsequently, the second strand was synthesized by adding 0.5 μl of 10X NEB buffer2 (NEB), 0.125 μl of dNTP mix (10 mM each nt; NEB), 0.15 μl of the Klenow fragment (3′ > 5′ exo-5,000 U/ml; NEB), 0.4 μl of second-NSR primer for mouse (100 μM; SIGMA custom DNA oligos), and 0.825 μl of distilled water (DW), followed by incubation for 60 min at 16°C and 10 min at 70°C. The double-stranded cDNA (ds-cDNA) was purified using AMPure XP beads (Beckman Coulter) and subjected to Tn5 Tagmentation in a 5 μl reaction with a homemade Tn5 enzyme, prepared as previously described (Picelli et al., 2014; Hennig et al., 2018). pTXB1-Tn5 (plasmid #60240; Addgene) was a gift from Rickard Sandberg (Karolinska Institutet, Stockholm, Sweden). After dissociating the Tn5 protein by adding SDS (0.04%), tagmented DNA was amplified through 17 PCR cycles using indexed primers and KAPA HiFi DNA Polymerase (Roche). Libraries were purified using AMPure XP beads, quantified by MultiNA (SHIMADZU), and pooled for next-generation sequencing on Illumina HiSeq 2500, HiSeq X, NovaSeq 6000, and MGI DNBSEQ-G400.

### RamDA-seq data processing

Short reads (3.9 ± 1.6 [mean ± SD] million reads for cells passed quality control [QC]) were trimmed to 50 bp using fastx_trimmer, if necessary (FASTX Toolkit v0.0.14, https://hannonlab.org/resources/). Then, low-quality parts and adapter sequences were trimmed using fastp (v0.20.0) (Chen et al., 2018) and mapped to the mm10 reference with STAR (v2.7.3a) (Dobin et al., 2013), utilizing a GTF file (GRCm38.99) obtained from https://www.ensembl.org. Unmapped reads and reads mapped with low-quality scores (MAPQ < 5) were removed using SAMtools (v1.9) (Li et al., 2009), resulting in the isolation of 73 ± 8.3% (mean ± SD) of total reads. Duplicated reads (40 ± 11.8%) were then filtered out using Picard Tools (v2.21.1) (https://broadinstitute.github.io/picard/). Mapped reads on each gene were counted using htseq-count (v0.11.2) (Anders et al., 2015) with the --stranded = no option and a GTF file from EMBL-EBI (GRCm38.99). Only cells with >200,000 reads mapped to over 8,000 genes were retained to exclude low-quality cells from the analysis. Expressions per cell were normalized by dividing the counts by the total counts for each cell, multiplying by 10,000, and then natural-log–transforming using log (+1) with the R package Seurat (Table S2). We employed the GeTMM (gene length corrected trimmed mean of M values) method by Smid et al. (2018) to normalize gene expression, using the edgeR package in R (Robinson et al., 2010), and gene length data were obtained from EMBL-EBI where indicated to investigate gene expression levels while minimizing the influence of transcriptome length.

### RamDA-seq data analysis and visualization

We selected the 6,000 most variably expressed genes using the FindVariableFeatures function and assigned 15 clusters among 1,980 QC-passed cells employing a graph-based clustering approach with the FindNeighbors and FindClusters functions in Seurat. The t-distributed stochastic neighbor embedding (t-SNE) representation was used for visualizing cell clusters and gene expressions (van der Maaten and Hinton, 2008). Normalized gene expressions in the log scale were standardized by scaling, and their mean values were used to evaluate the expression of a set of genes in individual cells. Differential gene expression between two groups of cells was assessed using a Wilcoxon rank-sum test in Seurat, and P values were adjusted for multiple comparisons by applying the Bonferroni correction. Genes expressed in <5% of cells in both groups were excluded from the analysis. When comparing multiple clusters, we excluded genes not differentially expressed in any clusters (adjusted P value <0.05; Benjamini–Hochberg-adjusted P values obtained by the fast Wilcoxon rank-sum test). For heatmap representations of gene sets for groups of cells, the mean expression of a gene within each cell group was normalized using z-score standardization across the plotted groups. Gene expressions were normalized to the 97.5th percentile value among the analyzed populations, facilitating visualization of expressions in individual cells. A gene was regarded as expressed if its expression in a cell was >25% of the 97.5th percentile. We calculated Pearson’s correlation coefficient between gene expressions using a designated set of cells to investigate coregulated genes and infer the underlying regulatory mechanism within specific cell populations. Specifically, we analyzed 463 cells from RamDA/c2 and c3 to examine 588 of 683 Aire-DEGs up-regulated predominantly in scMulti-seq/c3 in an Aire-dependent manner, considering that RamDA/c3 represented the corresponding cluster for RamDA/c2 in Aire-KO mice and exhibited defective expression of these Aire-DEGs. We used 283 cells from RamDA-c7, RamDA-8, RamDA-11, RamDA-13, and RamDA-14 to explore 812 of 846 Aire-DEGs up-regulated in an Aire-independent manner, primarily in scMulti-seq-c15, scMulti-seq-17, scMulti-seq-10, scMulti-seq-19, scMulti-seq-11, scMulti-seq-16, and scMulti-seq-4. Genes were excluded from the correlation analysis if they did not have raw mapped reads greater than or equal to five in at least five cells among the selected population. We performed clustering by affinity propagation based on the gene–gene Pearson correlation without specifying the number of clusters utilizing the R package apcluster (Frey and Dueck, 2007). To visualize the genomic position for genes exhibiting highly correlated expressions (Pearson’s r > 0.6), we used the R package BioCircos (Cui et al., 2016).

### Cluster matching between different datasets

To estimate the corresponding clusters between those identified by scMulti-seq and RamDA-seq, signature genes for each reference cluster were determined using the auROC and the Wilcoxon P value based on the Gaussian approximation with the Presto R package. We selected the top 2,000 genes by the absolute value of (AUC −0.5) among genes with an adjusted P value below 0.05. These signature genes were then utilized to compute the Pearson correlation coefficient between a query cell and the reference cluster. We assigned each query cell to the reference cluster with the highest Pearson’s r, omitting the assignment and categorizing it as unassigned if highest Pearson’s r was 0.2 or lower. The proportion of the matched reference cluster for each cell within a query cluster was visualized as a dot plot using the R package ComplexHeatmap (Gu et al., 2016). A similar analysis was performed for clusters identified by scMulti-seq in this study and scRNA-seq using Chromium Single Cell 3′ Reagent Kits from our previous study (GSE155331) (Nishijima et al., 2022), employing the top 500 signature genes for each cluster identified by scRNA-seq. Cluster sizes in GSE155331 were computed as the proportion of the corresponding cluster among all cells and normalized based on WT mice to highlight the differences between WT and Aire-KO mice.

We used R package SciBet (Li et al., 2020) with 2,000 genes selected by the E-test for identifying matched clusters between scMulti-seq and RamDA-seq including EED-cKO and dKO.

### Western blot analysis for H3K27me3 in mTECs

mTEC^high^ and mTEC^low^ were isolated using BD FACSAriaIII into a tube containing nuclear isolation buffer (Nuclei EZ Lysis Buffer; Sigma-Aldrich containing cOmplete EDTA-free protease inhibitor cocktail; Roche). Then, cells were collected by centrifugation (6,000 × *g* for 10 min at 4°C), and lysed with Laemmli sample buffer by incubating for 5 min at 95°C. For western blot analysis, nuclear lysate from ∼5,000 cells was separated by SDS-PAGE (10–20% SuperSep Ace; Wako) and electrotransferred to PVDF membranes (Clear Blot Membrane-P plus; ATTO) using a semi-dry blotting system (HorizeBLOT 2M; ATTO). The membrane was blocked for 30 min with 0.5% skim milk in Tris-buffered saline with Tween-20 (TBS-T, 50 mM Tris-HCl, 150 mM NaCl, and 0.05% Tween-20, pH 7.6), followed by western blotting with anti-H3K27me3 Ab (Rabbit mAb C36B11; CST) and HRP-conjugated anti-rabbit IgG (Peroxidase AffiniPure Goat Anti-Rabbit IgG [H+L], Jackson ImmunoResearch). Signals were developed using enhanced chemiluminescent (ECL) substrates (ImmunoStar Zeta; Wako) and detected by Amersham Imager 680 (Cytiva) and accompanying analysis software. The membrane was re-probed with anti-H3 Ab (rabbit anti-histone H3 polyclonal Ab, Proteintech) and HRP-conjugated anti-rabbit IgG after being treated with WB Stripping Solution (Nacalai Tesque) to normalize the signal variation due to the potential difference in cell numbers.

### CUT&Tag library preparation

CUT&Tag libraries were constructed as previously reported by Kaya-Okur et al. (2019), employing spike-ins for normalizations (Greulich et al., 2021). Specifically, 10,000 mTEC^high^ and mTEC^low^ were sorted using FACSAriaIII and collected in a tube containing 100 μl of BAMBANKER (GC LYMPHOTEC), then frozen gradually using BICELL (Nihon Freezer), and stored at −80°C until library construction. For sample preparation, cell-capturing beads were prepared by incubating 5 μl of streptavidin-conjugated magnetic beads (Dynabeads MyOne Streptavidin T1; Invitrogen) with 20 μg/ml biotin-conjugated ConA (C2272; Sigma-Aldrich) and 20 μg/ml biotin-conjugated WGA (L5142; Sigma-Aldrich) in 100 μl of PBS for 30 min at room temperature. The beads were then washed and resuspended in 20 μl of binding buffer (20 mM HEPES, pH 7.5, 10 mM KCl, 1 mM CaCl_2_, and 1 mM MnCl_2_). Frozen cells preserved in BAMBANKER were quickly thawed and washed with PBS, and 10,000 293FT cells (Invitrogen) were added for spike-in. Cells were resuspended in 100 μl of wash buffer (20 mM HEPES, pH 7.5, 150 mM NaCl, 0.5 mM spermidine, and cOmplete EDTA-free protease inhibitor cocktail; Roche) and mixed with 20 μl of cell-capturing beads for a 30-min incubation. The cells were then isolated using a magnetic separator (DynaMag-2 Magnet; Invitrogen) and resuspended in 55 μl of Ab solution (1:100 diluted anti-H3K27me3; CST#9733 in 20 mM HEPES, pH 7.5, 150 mM NaCl, 0.5 mM spermidine, protease inhibitor cocktail, 0.05% digitonin; WAKO water-soluble digitonin, 2 mM EDTA, and 0.1% BSA). Of the cell suspension, 5 μl was reserved for input library preparation (described later), while the remaining 50 μl was incubated overnight at 4°C with agitation using a rotary mixer (NISSINRIKA NRC-20D). Following overnight incubation, cells were washed with Dig-wash buffer (0.05% digitonin, 20 mM HEPES, pH 7.5, 150 mM NaCl, 0.5 mM spermidine, and protease inhibitor cocktail) and further incubated in secondary Ab buffer (1:100 diluted anti-rabbit IgG; ROCKLAND 611-201-122 in Dig-wash buffer) at 4°C for 1 h with agitation. Subsequently, cells were washed three times with Dig-wash buffer and incubated in 100 μl of 8 μg/ml in-house pAG-Tn5 in Dig-300 buffer (20 mM HEPES, pH 7.5, 300 mM NaCl, 0.5 mM spermidine, 0.01% digitonin, and protease inhibitor cocktail) at room temperature for 1 h with agitation. In-house pAG-Tn5, a fusion of Protein A and Protein G IgG binding domains with Tn5 transposase, was generated utilizing pTXB1-Tn5 and employing standard molecular biology techniques following previous studies (Picelli et al., 2014; Hennig et al., 2018). Then, cells were washed three times with Dig-300 buffer, resuspended in 100 μl of Tagmentation buffer (Dig-300 buffer containing 10 mM MgCl_2_), and incubated at 37°C for 1 h for DNA Tagmentation. The reaction was stopped by adding EDTA, SDS, and DNase-free RNase A to final concentrations of 15 mM, 0.5%, and 0.05 mg/ml, respectively, and incubated at 37°C for 15-min mixing at 600 rpm (ThermoMixer, Eppendorf). Tagmented DNA was purified by proteinase K treatment (Proteinase K, recombinant, PCR Grade, Roche) and phenol–chloroform extraction. Then, DNA was amplified using indexed primers and KAPA HiFi DNA Polymerase (Roche) to construct an NGS library. The libraries were purified using 1.1× volume AMPure XP beads. For input library preparation, DNA was extracted from 5 μl cell suspension by proteinase K treatment followed by phenol–chloroform extraction. DNA Tagmentation was carried out employing an in-house Tn5 enzyme. Subsequently, Tn5 was dissociated by adding 0.04% SDS, and DNA was amplified by PCR using indexed primers and KAPA HiFi DNA Polymerase. The libraries were purified and size-selected with AMPure XP beads (×0.55 volume to remove large fragments, followed by ×1.0 volume to remove smaller fragments). Libraries were quantified using MultiNA (SHIMADZU) and pooled for next-generation sequencing on Illumina HiSeq X.

### CUT&Tag data processing and analysis

Low-quality parts and adapter sequences were trimmed from short reads using fastp (v0.20.0) and mapped to the mm10 and hg38 genomes with bowtie2 (v2.3.5) using the -X 1000 option (Chen et al., 2018; Langmead and Salzberg, 2012). Quality-filtered reads (RQC) were counted in each sample after removing reads of low mapping quality (MAPQ < 30), improperly paired reads, and duplicated reads with SAMtools (v1.9) (Li et al., 2009). The fractions of spike-in cells (FrSpike, 43.6% on average) were determined from input libraries based on RQC normalized by the effective genome size for mm10 and hg38 (2.49 × 10^9^ and 2.86 × 10^9^, respectively, derived from deepTools documentation: https://deeptools.readthedocs.io/en/develop/content/feature/effectiveGenomeSize.html). Peaks were identified from pooled CUT&Tag datasets using MACS2 (v2.1.4) (Zhang et al., 2008) with options --broad --broad-cutoff 0.05, and the number of reads overlapping the peaks (reads in peaks; RiP) was counted using bedtools (v2.29.0) (Quinlan and Hall, 2010). CUT&Tag assay efficiency (Eff; 36.6 ± 2.9%) was computed from the RiP divided by RQC (Eff = RiP/RQC) of spike-in samples. The absolute scaling factor (Sfabs) for each sample was calculated as Sfabs=1/[RQC (hg38+mm10)×(1−FrSpike)]×1/Eff. These Sfabs were converted into relative scaling factors (Sfrel) by normalizing them to the highest Sfabs among all samples (Sfrel=Sfabs(sample)/max(allSfabs)). Normalized bedgraph files were generated using bedtools by piling up fragments while applying the -scale Sfrel option and then converted into bigwig files using bdg2bw (https://gist.github.com/taoliu/2469050). Mean signals on defined regions were computed using the normalized bigwig files and the rtracklayer package in R (Lawrence et al., 2009).

### ChIP followed by sequencing

Previously published Aire ChIP-seq data from mTEC^high^ (GSE92597) by Bansal et al. (2017) were utilized for the analysis. Short reads were preprocessed using fastp (v0.24.0) (Chen et al., 2018) to remove low-quality segments and adapter sequences. Reads were subsequently aligned to the mm10 genome using bowtie2 (v2.5.4) (Langmead and Salzberg, 2012). Low mapping quality reads (MAPQ < 30) and duplicate reads were filtered out using SAMtools (v1.21) (Li et al., 2009). Peaks were identified with MACS3 (v3.0.2) (Zhang et al., 2008) using the parameters --keep-dup all -B --SPMR -q 0.01.

### Bulk RNA-seq library preparation

RNA-seq libraries were prepared by applying the RamDA-seq method for scRNA-seq (Hayashi et al., 2018). Specifically, mTEC^high^ (DAPI^−^CD45^−^EpCAM^+^Ly51^−^MHC-II^high^) and mTEC^low^ (DAPI^−^CD45^−^EpCAM^+^Ly51-MHC-II^low^) from 4- to 6-wk-old mice for WT and Aire-KO comparison, and 1- to 6-day-old mice for EED-cKO, Aire-KO, and Aire/EED-dKO, were sorted by FACSAriaIII into tubes containing lysis buffer (Buffer TCL; Qiagen). Total RNAs were isolated using RNAClean XP beads (Beckman Coulter) directly from the lysis buffer and eluted into 10 μl of water containing RNase inhibitor (4 U/μl RNasin Plus RNase Inhibitor; Promega). The RNA was denatured at 65°C for 5 min, placed on ice, and treated with DNase I (0.1 U/μl DNase I, Amplification Grade; Invitrogen) in 0.25x RT Buffer (PrimeScript RT Reagent Kit; Takara) at 30°C for 15 min. Then, cDNA was synthesized in 30 μl of 1x RT Buffer containing RT Enzyme Mix (1.5 μl of PrimeScript RT Enzyme Mix I; Takara), oligo(dT)-RT primer (0.2 μM Oligo[dT]18 Primer; Thermo Fisher Scientific), T4 gene 32 protein (0.033 mg/ml; NEB), and first-NSR primer for mouse (3.3 μM; SIGMA custom DNA oligos) by incubating for 10 min at 25°C, 10 min at 30°C, 30 min at 37°C, 5 min at 50°C, and 5 min at 94°C. The second strand was synthesized by adding 5 μl of 10X NEB buffer2 (NEB), 1.25 μl of dNTP mix (10 mM each nt; NEB), 1.5 μl of the Klenow fragment (3′>5′ exo- 5,000 U/ml; NEB), 5 μl of second-NSR primer for mouse (100 μM; SIGMA custom DNA oligos), and 7.25 μl of DW, and incubating at 16°C for 60 min followed by 10 min at 70°C. The ds-cDNA was purified using the AMPure XP beads (Beckman Coulter) and quantified using Qubit (Qubit dsDNA HS Assay Kit; Invitrogen). Then, ds-cDNA <1 ng was used for Tn5 tagmentation in a 25 μl reaction with a homemade Tn5 enzyme prepared as previously described (Picelli et al., 2014; Hennig et al., 2018). Tagmented DNA was purified using AMPure XP beads and amplified by 10–15 cycles of PCR using indexed primers and KAPA HiFi DNA Polymerase (Roche). The libraries were purified using AMPure XP beads and pooled for next-generation sequencing on Illumina HiSeq 2500, HiSeq X, and MGI DNBSEQ-G400.

### Bulk RNA-seq data processing and analysis

Short reads were trimmed to 50 bp using fastx_trimmer when necessary (FASTX Toolkit v0.0.14, https://hannonlab.org/resources/). Following the trimming of low-quality reads and adapter sequences by fastp (Chen et al., 2018), short reads were mapped to mm10 using STAR (2.7.3a) (Dobin et al., 2013), with an Ensembl GTF file for mm10 downloaded from https://www.ensembl.org. Unmapped reads and reads mapped with low-quality scores (mapping quality <5) were removed using SAMtools (v1.9) (Li et al., 2009), while duplicated reads were removed by Picard MarkDuplicates (https://broadinstitute.github.io/picard). Mapped reads for each transcript were counted using htseq-count (version 0.11.2) (Anders et al., 2015) with the --stranded = no option and an Ensembl GTF file for mm10. Raw read counts on transcripts were processed using the edgeR package (v3.20.9) (Robinson et al., 2010) in R for normalization and identification of differentially expressed genes (Table S2). We defined genes down-regulated in mTEC^high^ from Aire-KO compared with WT (FC < 0.5 and FDR < 0.05 for Aire-KO/WT) as bulk Aire-induced genes. In contrast, genes expressed comparably in mTEC^high^ from Aire-KO and WT (|log_2_(expFC)| < 0.2 and FDR > 0.05) and in WT/c3 and Aire-KO/c1 by scMulti-seq (adj. P value ≥ 1 × 10^−5^) were classified as Aire-neutral genes.

### Cell culture and transfection

293FT cells (Invitrogen) were cultured in DMEM (High Glucose) with L-glutamine (Wako) supplemented with 10% (vol/vol) FBS and penicillin–streptomycin antibiotics (Wako) and maintained in a humidified atmosphere at 37°C with 5% CO_2_. For transfection, around 3.5 × 10^6^ cells were seeded in 10-cm tissue culture plates a day before and transfected with pCMV-Tag1 plasmid (Clontech) carrying Flag-tagged murine Aire, employing TransIT-293 Reagent (Mirus) according to the manufacturer’s instructions.

### Coimmunoprecipitation (Co-IP) and western blot analysis using transfected cells

Co-IPs followed by western blot analysis were performed as previously described (Yoshida et al., 2015). Specifically, about 48 h after the transfection, 293FT cells were harvested after washing twice with cold PBS, resuspended in hypotonic buffer (20 mM Tris-HCl, pH 7.5, 3 mM MgCl_2_, 10 mM NaCl, 1 mM DTT, cOmplete EDTA-free protease inhibitor mixture; Roche, and Halt Phosphatase Inhibitor Cocktail; Thermo Fisher Scientific), and kept on ice for 20 min. Then, the cell suspension was centrifuged at 400 × *g* for 5 min at 4°C after adding NP-40 (0.1%) and vortexing for 5 s to obtain nuclear fraction. The nuclear pellet was resuspended in a nuclease digestion buffer (250 U/ml Universal Nuclease for Cell Lysis; Pierce, 20 mM Tris-HCl, pH 8.0, 0.1% NP-40, 2 mM MgCl_2_, 10 mM NaCl, 1 mM DTT, and 10% glycerol) and incubated at room temperature for 15 min. Then, EDTA and NaCl were added to a final concentration of 5 and 420 mM, respectively, and incubated for 1 h at 4°C under rotation. Subsequently, the suspension was homogenized using a glass Dounce tissue grinder with 10 up-and-down strokes by the tight pestle to release the nuclear proteins and centrifugation at 13,000 × *g* for 10 min at 4°C. The supernatant was isolated and used as a nuclear extract for IP or co-IP experiments.

For an IP, 2 μg of anti-Flag Ab (monoclonal ANTI-FLAG M2 Ab produced in mouse; Sigma-Aldrich) or control mouse IgG (Mouse IgG Isotype Control; Abcam) was added, and the nuclear extract was incubated on a rotator overnight at 4°C. Then, 20 μl of Dynabeads Protein G (VERITAS) was added before incubating for another 1 h. The Dynabeads were washed four times with ice-cold PBS containing 0.1% NP-40. IPed proteins were eluted by boiling in sample buffer for 5 min, separated on a 10–20% SDS gel, and then transferred to a PVDF membrane (Immobilon-P PVDF 0.45 μm; Millipore) for western blot analysis. For sequential detection of different targets, blots were incubated in WB Stripping Solution (Nacalai Tesque) for 15 min before the subsequent blocking. The following Abs were used: anti-Ezh2 (rabbit mAb D2C9; CST), anti-Suz12 (rabbit mAb D39F6; CST), anti-H3K27Ac (mouse monoclonal IgG1, clone CMA309; Millipore), anti-H3K27me3 (rabbit mAb C36B11; CST), anti-Flag (rabbit mAb D6W5B; CST), HRP-conjugated anti-rabbit IgG, and HRP-conjugated anti-mouse IgG (anti-mouse Ig HRP, TrueBlot ULTRA; ROCKLAND). Signals were developed using ECL substrates and detected by Amersham Imager 680 and accompanying analysis software.

### Gel filtration analysis of PRC2

The preparation of nuclear extracts and gel filtration analysis was performed as described previously (Casanova et al., 2011; Chen et al., 2020). Specifically, to prepare nuclear extracts from Aire- or control-transfected 293FT cells, cells were harvested from two 10-cm dishes ∼48 h after transfection, following two washes with cold PBS. Cells collected by centrifugation were resuspended in hypotonic buffer (0.1% Triton X-100, 10 mM HEPES, pH 7.5, 10 mM KCl, 1.5 mM MgCl_2_, cOmplete EDTA-free protease inhibitor mixture; Roche, and Halt Phosphatase Inhibitor Cocktail; Thermo Fisher Scientific) and incubated on ice for 20 min. Then, nuclei were isolated by vortexing for 5 s after adding Triton X-100 (0.1%), collected by centrifugation at 14,000 × *g* at 4°C for 30 s, and resuspended in digestion buffer (250 U/ml Pierce Universal Nuclease for Cell Lysis; Thermo Fisher Scientific, 20 mM HEPES, pH 7.5, 0.1% NP-40, 1 mM MgCl_2_, 3 mM CaCl_2_, 20 mM KCl, cOmplete EDTA-free protease inhibitor mixture; Roche, and Halt Phosphatase Inhibitor Cocktail; Thermo Fisher Scientific, and 10% glycerol). Then, the suspension was incubated at room temperature for 15 min, followed by another incubation at 4°C for 30 min on rotation after adding NaCl (300 mM) and EDTA (10 mM). Subsequently, nuclear extracts were collected by centrifugation at 13,000 × *g* at 4°C for 5 min and filtered through a hydrophilic PVDF membrane filter (0.65 μm pore size, Ultrafree-MC Centrifugal Filter; Merck) before subjecting to a SEC. The SEC analysis was performed by injecting the nuclear extract at a flow rate of 0.4 ml/min into a Superose6 10/300 Gl column (Cytiva) in SEC buffer (0.1% NP-40, 20 mM HEPES, pH 7.5, 150 mM NaCl, 20 mM KCl, and 1 mM EDTA) connected to AKTA pure chromatography system (Cytiva). Fractions of 500 μl were collected and analyzed by western blotting using the following Abs: anti-Ezh2 (rabbit mAb D2C9; CST), anti-Flag (mouse mAb M2; Sigma-Aldrich), HRP-conjugated anti-rabbit IgG (Peroxidase AffiniPure Goat Anti-Rabbit IgG (H+L); Jackson ImmunoResearch), and HRP-conjugated anti-mouse IgG (Peroxidase AffiniPure Goat Anti-Mouse IgG (H+L); Jackson ImmunoResearch). Signals were developed using ECL substrates (ImmunoStar Zeta; Wako) and detected by Amersham Imager 680 and accompanying analysis software. The quantified signals were normalized based on the median value in high molecular weight (MW) fractions (MW > 670 kDa) to assess EZH2 abundance in each fraction. The relative abundance of Ezh2 in the fractions of equivalent MW from Aire- or control-transfected 293FT cells was computed, and their means in high MW (>670 kDa) and low MW (670–150 kDa) were employed to represent the relative abundance of EZH2 in high-MW and low-MW fractions. Before the assay, the column was pre-calibrated using the protein standards containing thyroglobulin (670 kDa), gamma globulin (150 kDa), ovalbumin (44.3 kDa), ribonuclease A (13.7 kDa), and p-aminobenzoic acid (Protein Standard Mix 15–600 kDa; Supelco).

### Statistical analysis

Statistical significance was evaluated utilizing suitable statistical tests. The following notations were used in all relevant panels: P = ns, not significant; *P < 0.05; **P < 0.01; ***P < 0.001. Statistical analyses were conducted using R and GraphPad Prism 7 (GraphPad Software).

### Online supplemental material

Fig. S1 shows the evaluation of scMulti-seq and characterization of identified TEC clusters. Fig. S2 shows the evaluation of RamDA-seq and demonstration of two types of Aire-DEGs (Aire-driven genes and Aire-enhanced genes). Fig. S3 shows integrative analyses of scMulti-seq data with conventional scRNA-seq (Nishijima et al., 2022) and bulk RNA-seq. Fig. S4 shows detailed profiles of mTECs from various genotypes and Aire-transfected cells: FACS analysis, western blotting, and SEC. Fig. S5 shows RamDA-seq analyses employing EED-cKO and Aire/EED-dKO. Table S1 is the list of supplemental data related to scMulti-seq. Table S2 is the list of supplemental data related to RamDA-seq. Table S3 is the list of supplemental data related to bulk RNA-seq.

## Supplementary Material

Table S1 lists supplemental data related to scMulti-seq.

Table S2 lists supplemental data related to RamDA-seq.

Table S3 lists supplemental data related to bulk RNA-seq.

SourceData F5 is the source file for Fig. 5.

SourceData FS4 is the source file for Fig. S4.

## Data Availability

The datasets associated with this study have been deposited at the Gene Expression Omnibus (GEO) under the following accession numbers: GSE205560 for scMulti-seq, GSE205991 and GSE249591 for RamDA-seq, GSE205410 for bulk RNA-seq, and GSE206236 for CUT&Tag. We utilized publicly available programs and scripts as specified in each analysis and did not generate original code. Any additional information required to reanalyze the data reported in this paper can be obtained upon request by contacting H. Yoshida.
