# Supplementary figures and images for: Role of PRC2 in the stochastic expression of Aire target genes and development of mimetic cells in the thymus
Source: J Exp Med. 2025 Apr 17;222(7):e20240817. doi: 10.1084/jem.20240817 (PMC12005117; doi:10.1084/jem.20240817)

5F

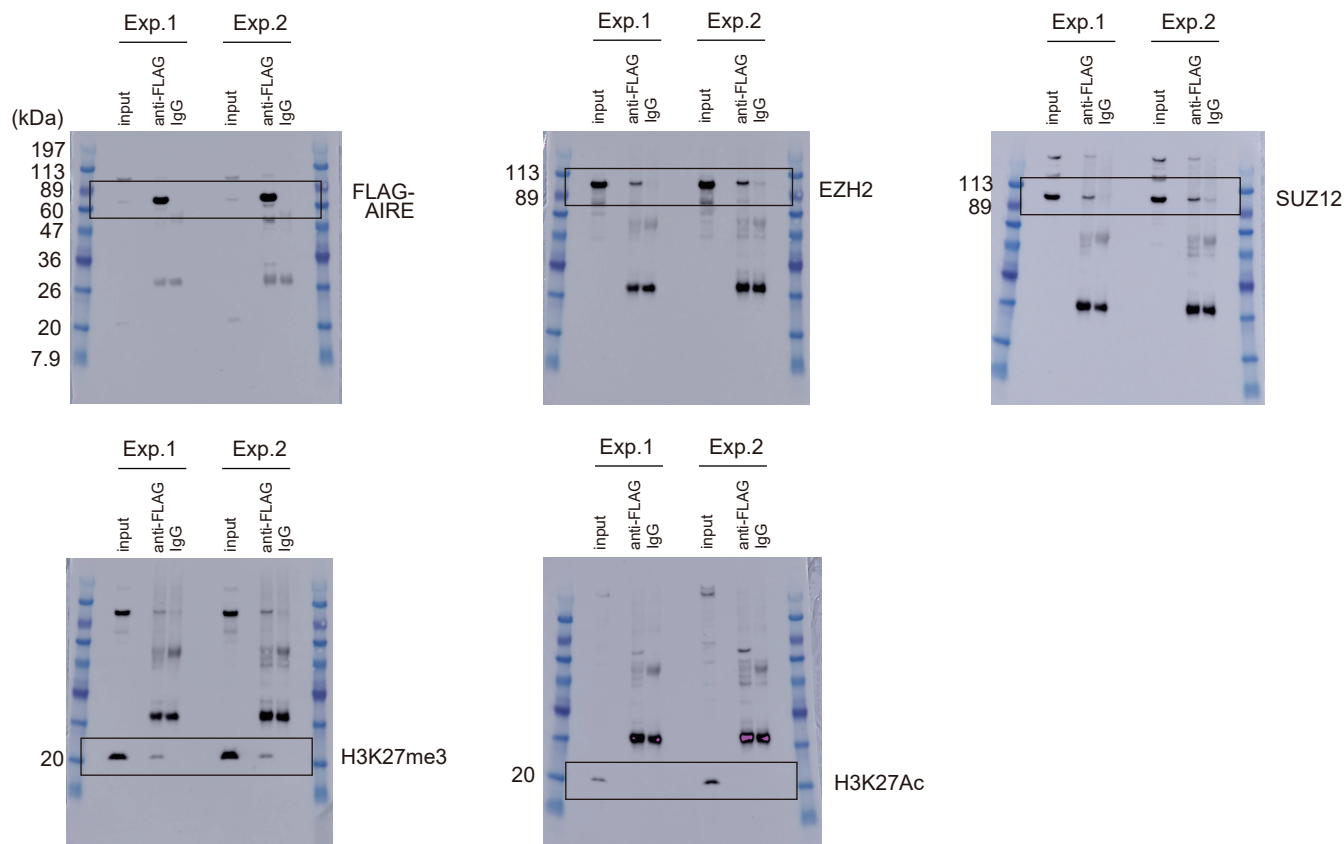

5G

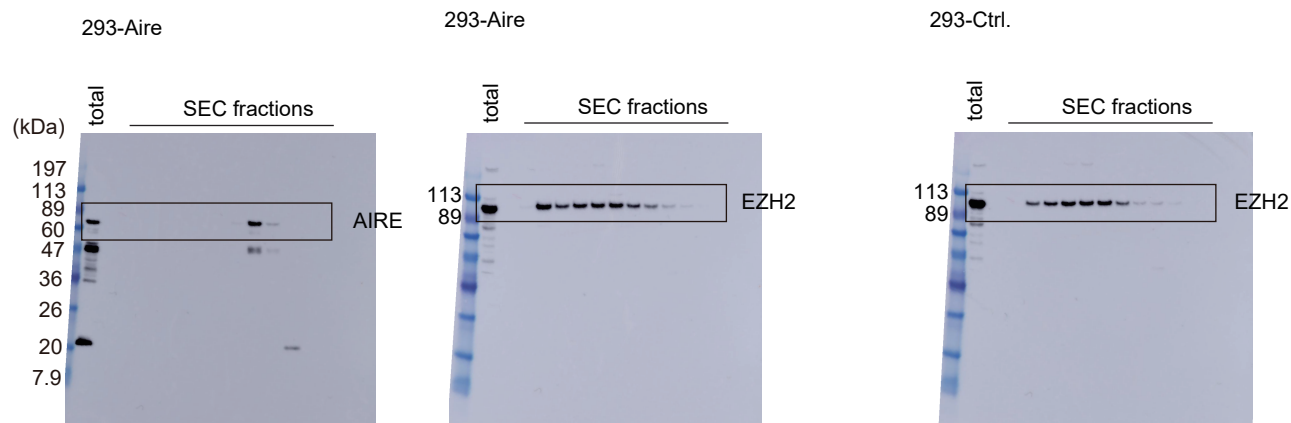

Supplement: SourceData F5 — is the source file for Fig. 5. [file jem_20240817_sourcedataf5.pdf]

S4B

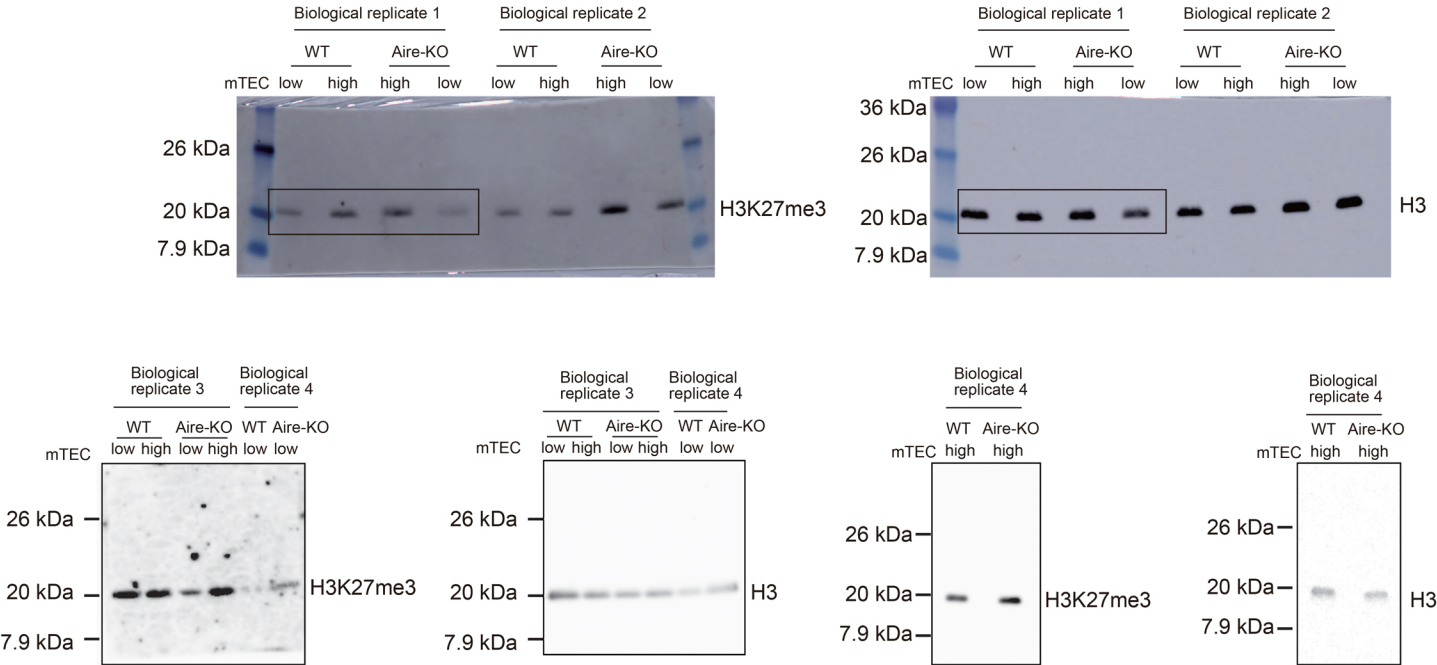

S4E

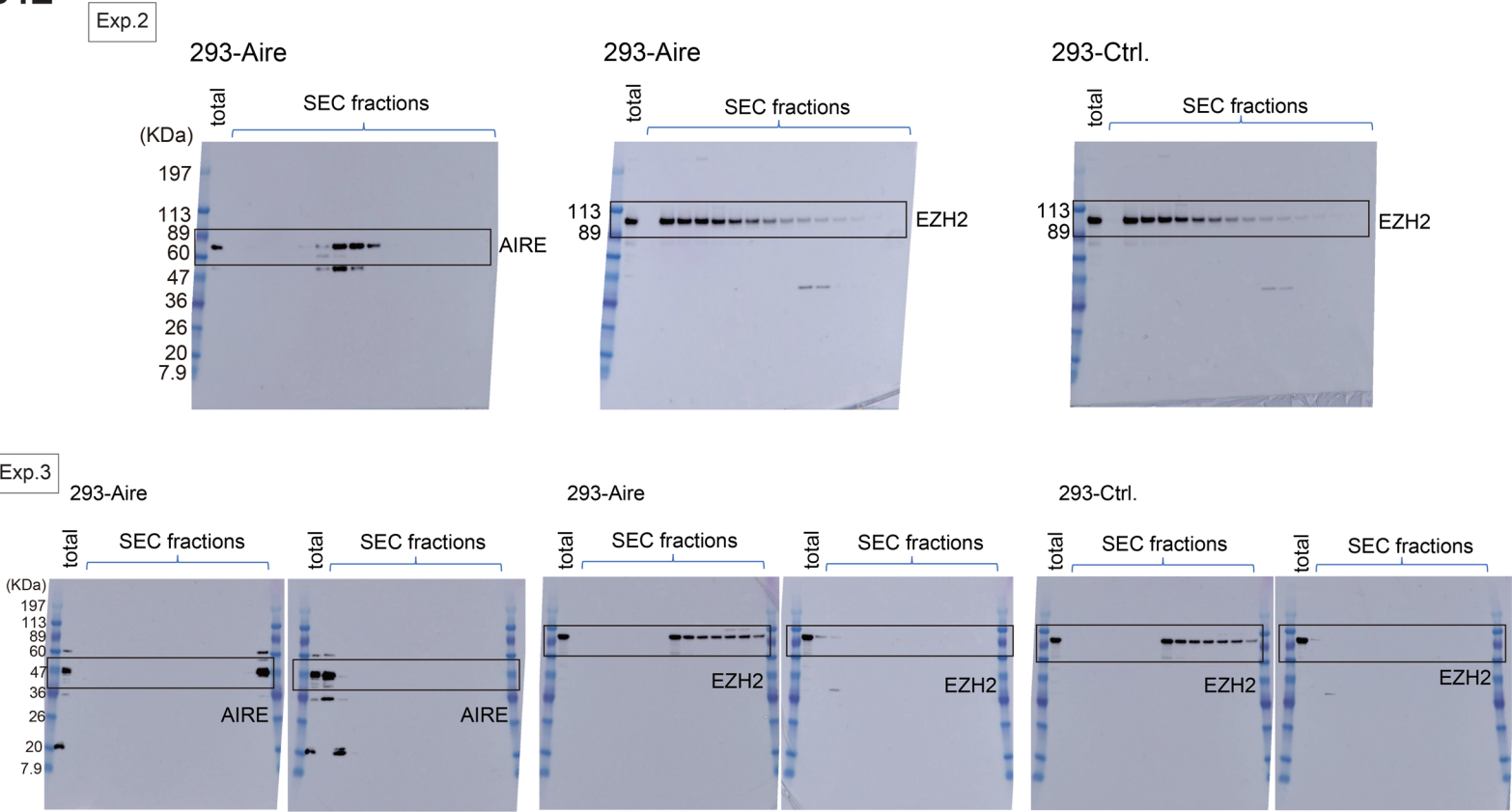

Supplement: SourceData FS4 — is the source file for Fig. S4. [file jem_20240817_sourcedatafs4.pdf]
